# A Systematic Comparison of Linear Regression–Based Statistical Methods to Assess Exposome-Health Associations

**DOI:** 10.1289/EHP172

**Published:** 2016-05-24

**Authors:** Lydiane Agier, Lützen Portengen, Marc Chadeau-Hyam, Xavier Basagaña, Lise Giorgis-Allemand, Valérie Siroux, Oliver Robinson, Jelle Vlaanderen, Juan R. González, Mark J. Nieuwenhuijsen, Paolo Vineis, Martine Vrijheid, Rémy Slama, Roel Vermeulen

**Affiliations:** 1 Team of Environmental Epidemiology, Inserm, CNRS, University Grenoble-Alpes, IAB (institute for Advanced Biosciences), Grenoble, France; 2 Institute for Risk Assessment Sciences, Utrecht University, Utrecht, the Netherlands; 3 Department of Epidemiology and Biostatistics, MRC-PHE (Medical Research Council–Public Health England) Centre for Environment and Health, School of Public Health, Imperial College London, London, United Kingdom; 4 ISGlobal, Center for Research in Environmental Epidemiology (CREAL), Barcelona, Spain; 5 Universitat Pompeu Fabra (UPF), Barcelona, Spain; 6 CIBER Epidemiología y Salud Pública (CIBERESP), Madrid, Spain

## Abstract

**Background:**

The exposome constitutes a promising framework to improve understanding of the effects of environmental exposures on health by explicitly considering multiple testing and avoiding selective reporting. However, exposome studies are challenged by the simultaneous consideration of many correlated exposures.

**Objectives:**

We compared the performances of linear regression–based statistical methods in assessing exposome-health associations.

**Methods:**

In a simulation study, we generated 237 exposure covariates with a realistic correlation structure and with a health outcome linearly related to 0 to 25 of these covariates. Statistical methods were compared primarily in terms of false discovery proportion (FDP) and sensitivity.

**Results:**

On average over all simulation settings, the elastic net and sparse partial least-squares regression showed a sensitivity of 76% and an FDP of 44%; Graphical Unit Evolutionary Stochastic Search (GUESS) and the deletion/substitution/addition (DSA) algorithm revealed a sensitivity of 81% and an FDP of 34%. The environment-wide association study (EWAS) underperformed these methods in terms of FDP (average FDP, 86%) despite a higher sensitivity. Performances decreased considerably when assuming an exposome exposure matrix with high levels of correlation between covariates.

**Conclusions:**

Correlation between exposures is a challenge for exposome research, and the statistical methods investigated in this study were limited in their ability to efficiently differentiate true predictors from correlated covariates in a realistic exposome context. Although GUESS and DSA provided a marginally better balance between sensitivity and FDP, they did not outperform the other multivariate methods across all scenarios and properties examined, and computational complexity and flexibility should also be considered when choosing between these methods.

**Citation:**

Agier L, Portengen L, Chadeau-Hyam M, Basagaña X, Giorgis-Allemand L, Siroux V, Robinson O, Vlaanderen J, González JR, Nieuwenhuijsen MJ, Vineis P, Vrijheid M, Slama R, Vermeulen R. 2016. A systematic comparison of linear regression–based statistical methods to assess exposome-health associations. Environ Health Perspect 124:1848–1856; http://dx.doi.org/10.1289/EHP172

## Introduction

Environmental factors comprise a wide range of physical, chemical, biological, and sociological stressors. As exemplified in twin and migrant studies, the environment may explain a relatively large fraction of the variation in the risk of many chronic diseases or continuous health traits (Rappaport et al. 2014; Willett 2002). Until now, studies in environmental epidemiology typically assessed the link between environmental exposures and health using approaches considering each environmental exposure separately; therefore, they provided only a fragmented view of environment and health associations (Buck Louis et al. 2013; Rappaport 2011; Vrijheid et al. 2014; see Greenland 1994; Lenters et al. 2015 for exceptions). Results from these approaches suffer from possible confounding caused by (ignored) coexposures, selective reporting, and publication bias (Patel and Ioannidis 2014; Slama and Vrijheid 2015). The exposome concept, as originally defined by Wild (2005), comprises the totality of environmental exposures from the prenatal period onwards and argues for a holistic consideration of all exposures simultaneously (Wild 2012).

Most previous studies relating the exposome to health relied on an environment-wide association study (EWAS, the association between each single exposure factor and the outcome being estimated separately) (Patel et al. 2010), which was sometimes followed by a multiple regression step that included the selected predictors (Patel et al. 2013). Several multivariate regression–based statistical methods are now well established and allow accounting for a potential joint action of multiple exposures on health (Chadeau-Hyam et al. 2013). Sparse partial least squares (sPLS; Chun and Keleș 2010), for instance, has recently been used in a study of male fecundity (Lenters et al. 2015), and elastic net (ENET; Zou and Hastie 2005) was used to link multiple environmental contaminants to birth weight (Lenters et al. 2016). To our knowledge, in the context of exposome research, no other multiple regression statistical method has yet been applied.

The performances of these established statistical methods in an exposome context remain to be systematically assessed. In a recent simulation study (Sun et al. 2013), several multiple regression approaches were investigated for a limited number of exposures (*n* ≤ 20) that were, at most, moderately correlated (Pearson correlation < 0.57). However, in (future) exposome studies, many more covariates will likely be considered, and stronger correlations (typically > 0.6) are routinely observed in large exposome datasets, such as NHANES (Patel et al. 2010, 2013; Patel and Ioannidis 2014). We therefore extended the work by Sun et al. to a realistic exposome context and aimed to compare the statistical performances of linear regression–based statistical methods for future exposome studies.

We generated exposure data using an empirical correlation structure between a large number of exposure covariates (i.e., 237) and assumed that 0–25 of these exposures linearly influenced a continuous health outcome without effect measure modification (i.e., interaction). The statistical methods that we compared were *a*) the EWAS approach; *b*) EWAS followed by a multiple regression step including the identified hits; *c*) ENET, a penalized regression method; *d*) sPLS regression, a supervised dimension reduction method; *e*) the Graphical Unit Evolutionary Stochastic Search (GUESS) algorithm, a computationally optimized Bayesian variable selection method (Bottolo et al. 2013); and *f*) the deletion/substitution/addition (DSA) sequential algorithm (Sinisi and van der Laan 2004). The statistical performances of the selected approaches were systematically compared on the basis of six established criteria and two modified criteria to evaluate both variable selection and point estimation. We also investigated the sensitivity of the statistical performances of the methods with respect to modifications of the empirical correlation structure used to generate the exposures.

## Methods

Our simulation model relied on generating a matrix of exposure variables *X* for a fictitious population. From this matrix, we generated the health outcome *Y* according to a linear regression model; seven scenarios were defined on the basis of the number of true predictors. We assessed the association between each simulated *X* and *Y* using a preselected set of statistical methods whose performances were assessed for each scenario and were compared using the metrics detailed below. For each scenario, we simulated 100 independent data sets.

### Generation of the Exposome

To generate exposure variables with a realistic correlation structure, we relied on the existing INMA (Infancia y Medio Ambiente) mother-child cohort (Guxens et al. 2012), in which a total of 237 environmental factors were assessed in mothers during pregnancy through questionnaires, geospatial modeling, and biological monitoring. From the matrix of all pairwise correlations, we computed the closest positive definite matrix (Higham 2002) and used this estimate as our benchmark correlation matrix ∑ (see Figure S1). We used Σ to generate *X*, the exposome of a virtual study population of 1,200 subjects [size of the study population of an ongoing European exposome project comprising the INMA cohort (Vrijheid et al. 2014)], from a mean-centered multivariate normal distribution: *X* ~ *N*(*0,* Σ), where *N* is the multivariate Gaussian distribution. As the cohort data contained five binary variables (the others were continuous), we have dichotomized these five variables in our simulated data sets to replicate the proportion of positive responses observed in the original data.

### Health Outcome Generation

The health outcome *Y* was generated as a function of the exposome according to


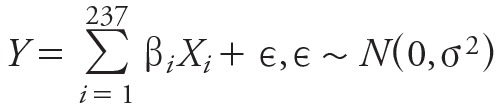
[1]

where *X_i_* is the vector containing all values for predictor *i* and where *e* represents the residuals of the regression model. Regression coefficients β*_i_* were all set to 0 except for the *k* randomly selected variables that were assumed to be causally related to the outcome (hereafter referred to as “true predictors”), for which β*_i_ =* 1. We considered seven scenarios, each defined by a different number of true predictors: *k* = 0, 1, 2, 3, 5, 10, and 25. The residual variance σ^2^ was defined such that the proportion of variance explained by the true predictors (*R* ^2^) equaled 3% × *k*. With this constraint, the signal to noise ratio was the same in all simulations within a given scenario, and the power to select a true predictor in unadjusted analyses with uncorrelated true predictors was constant across scenarios (see Supplemental Material S1).

Seven versions of these scenarios were generated. Set 1 corresponds to the scenarios defined above. Sets 2 and 3 aimed to assess the impact of the correlation level amongst true predictors, which could raise identifiability issues. These scenarios differed from set 1 by ensuring that correlation among all true predictors was in absolute value < 0.2 for set 2, and > 0.5 for set 3. Sets 4 and 5 aimed to assess the impact of the correlation structure of the whole exposome on the performances of the statistical methods; the scenarios differed from set 1 by not generating *X* from Σ, but for set 4 from the correlation matrix Σ^–^ that was obtained by dividing the coefficients of Σ by 2 except on the diagonal; and for set 5 from Σ^+^ that was obtained by multiplying the coefficients of Σ by 2, upper-bounding the coefficients by 1, and computing the closest semidefinite matrix. Set 6 investigated deviating from the assumption of normally distributed exposures (i.e., including potentially skewed distributions and outliers) by generating scenarios similarly to set 1 except with exposure data obtained by bootstrapping the actual environmental data from the INMA cohort. Finally, set 7 investigated the methods’ robustness to unequal effect sizes by generating scenarios similarly to set 1 except with effect sizes (i.e., β*_i_*) for true predictors drawn from a uniform distribution in [0.5, 1.5].

In all scenarios, the health outcome was generated as described above; for a given number of true predictors, the proportion of the variance explained by the true predictors was therefore the same across all seven sets of scenarios.

### Statistical Methods to Estimate the Exposome-Health Association

To estimate the association between *Y* and *X*, we used six linear regression–based statistical methods.

***Environment-wide association study.*** The EWAS (Patel et al. 2010) relies on linear regression models fitted independently for each covariate. The statistical significance of the association between predictors and the response is assessed on the related two-sided *p-*values after a correction for multiple comparisons is applied. As a benchmark, we considered the widely used Benjamini and Yekutieli (2001) correction to control the false discovery rate (FDR) at a desired level (here, 5%). Additionally, covariates declared significant in the EWAS were included in a multiple linear regression model and were retained if their two-sided *p-*value was below 5% (Tzoulaki et al. 2012). This two-step approach is referred to as EWAS-multiple linear regression (EWAS-MLR).

As sensitivity analyses, we tested several procedures to correct for multiple hypothesis testing: a permutation-based approach (Patel et al. 2010), the Benjamini and Hochberg (1995) procedure, and the Bonferroni (1936) correction. We also tested the EWAS method without applying a correction for multiple comparisons as a way to illustrate what would happen if independent studies were separately performed for each exposure covariate.

***Elastic net.*** The ENET (Zou and Hastie 2005) is a penalized regression model that relies on a generalized linear framework, and it uses a weighted mixture of the least absolute shrinkage and selection operator (LASSO) (Tibshirani 1996) and ridge (Hoerl and Kennard 1970) penalties. The LASSO penalty promotes sparsity and performs variable selection through shrinkage: the lowest regression coefficients, corresponding to the least informative predictors, are attributed a zero value. The ridge penalty accommodates correlated variables and ensures numerical stability. The calibration of the tuning parameters, the overall penalty, and the mixing proportion for the two penalties were determined by minimizing the prediction root mean squared error (RMSE) using 10-fold cross-validation (i.e., the data were partitioned into 10 subsets; for each of these subsets, the data were trained on the other 9 partitions and fitted on the given left-out subset, over which the RMSE was estimated). To prevent over-fitting, the optimal calibration parameters were defined as those providing the most sparse model (as measured by the number of nonzero regression coefficients) among those yielding an RMSE within 1 standard error of the minimum RMSE (Meinshausen and Bühlmann 2006).

***Sparse partial least squares regression.*** Partial least squares regression is a supervised dimension reduction technique that builds summary variables as linear combinations of the original set of variables. To ensure that the resulting lower-dimension representation of the data is relevant to the outcome of interest, the components are defined iteratively such that they explain as much of the remaining covariance between the predictors and the (health) outcome as possible. The sPLS approach simultaneously yields good predictive performance and appropriate variable selection by creating sparse linear combinations of the original predictors (Chun and Keleş 2010). Sparsity is induced by including a penalty (η) in the estimation of the linear combination coefficients; that is to say, all coefficients with an absolute value lower than some fraction η of the maximum absolute coefficient are shrunk to zero. This procedure is called soft thresholding (Lenters et al. 2015). Only the first *K* components are included as covariates in a linear regression model. The values of *K* and η were calibrated by minimizing the RMSE using 5-fold cross-validation (the default implementation). To complete the model comparison, we generalized the reference implementation such that it also included the empty model (*K* = 0).

***Graphical Unit Evolutionary Stochastic Search.*** As part of the Bayesian variable selection approaches, GUESS seeks models that optimally predict the health outcome. Each model is defined by a unique combination of covariates (Bottolo and Richardson 2010). Method estimation requires identification of the most relevant models among the 2*^p^* (where *p* denotes the total number of covariates) possible combinations of covariates using an evolutionary Monte Carlo algorithm, which combines tempered multiple chains run together with genetic algorithms. This Monte Carlo algorithm ensures both improved mixing of the sampler and exchange of information across chains (Bottolo et al. 2013).

For each simulated data set, we ran the GUESS algorithm for 20,000 iterations and discarded the first 5,000 to account for burn-in. We set the number of chains to 3. To ease convergence and to prevent extensive parameter calibration, noting *E* (the *a priori* expected model size) and ρ (its variance), we set *E* = 3 and ρ = 3 for *k* < 5, and *E* = *k* + 2 and ρ = 5 for *k* ≥ 5. As a conservative measure, among the models visited, we retained those associated with a posterior probability > 0.01.

From the union of all exposures included in these models retained, we selected those with a marginal posterior probability of inclusion (MPPI; the probability that a variable is included in any of the models retained) greater than the (1 – 0.05/237) quantile of the MPPI distribution under the null hypothesis (i.e., where no covariate was associated with the outcome).

The original goal of GUESS was to select the best combination(s) of covariates to predict the outcome. Its latest implementation (Liquet et al. 2016) allows posterior simulation of the coefficients’ estimates for a given model. However, in our simulation context, where the true predictors are different from one data set to another, this indirect (i.e., conditional on variable selection) estimation procedure would require integrating posteriors over all models visited, which represents a prohibitive computational effort and is therefore incompatible with a direct coefficient estimation. As a conservative alternative, we used an additional ridge regression step with the variables selected by GUESS to estimate the methods’ coefficients. However, this procedure is likely to lower the quality of the estimates.

***Deletion-Substitution-Addition algorithm.*** DSA is an iterative linear regression model search algorithm (Sinisi and van der Laan 2004). The set of potential models is limited by three user-specified constraints: the maximum order of interaction amongst predictors, the maximum power for a given predictor, and the maximum model size. At each iteration, the following three steps are allowed: *a*) removing a term, *b*) replacing one term with another, and *c*) adding a term to the current model. The search for the best model starts with the intercept model and identifies an optimal model for each model size. The final model is selected by minimizing the value of the RMSE using 5-fold cross-validated data. We allowed no polynomial or interaction terms, and we considered models including up to 40 covariates (however, this number was never reached in our simulations).

We used R implementations of the statistical methods under investigation, which are available in the packages stats, glmnet, spls, R2GUESS and DSA, respectively (version 3.1.1, R Project for Statistical Computing; version 2.15.3 for DSA). The R codes developed by the authors and the correlation matrix Σ are provided in Supplemental Material S2 and Excel File Table S1, respectively.

### Statistical Performance Assessment

The performances of each statistical method were evaluated using key criteria that measured the relevance of the variable selection and the quality of the point estimates.

The sensitivity of a method was calculated for each scenario and simulation as the proportion of true predictors that were actually selected by the given method. The specificity was calculated in the same way as the proportion of unrelated exposures that were not selected.

The false discovery proportion (FDP) was defined as the proportion of selected variables that were not genuinely related to the outcome. When no variable was selected in a given run, we considered that no variable was mistakenly selected, and the FDP was given a value of 0%. The FDP and the sensitivity were not computed for scenarios with 0 true predictors.

We investigated the accuracy of the estimated coefficients using the mean absolute bias calculated over the 237 coefficient estimates as


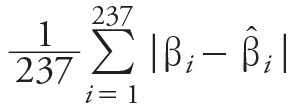
[2]

where β*_i_* represents the coefficient used in the simulation, and

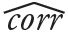
 represents the corresponding estimate. The mean absolute bias was also separately computed over the true predictors and over the unrelated exposures (i.e., non-true predictors).

Owing to the possibly strong correlations between exposures, the argument could be made that not selecting a true predictor but instead picking up another highly correlated variable should not be seen as a complete false selection, in the sense that the statistical method did not completely miss the signal. To account for this viewpoint, in our study, we defined alternative sensitivity and FDP measures accounting for such a partial agreement based on the highest absolute correlation estimated between the true predictors and the covariates selected by the statistical method:


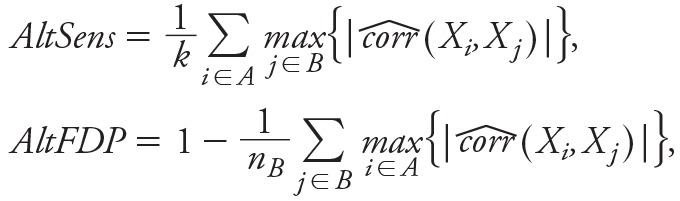
[3]

where *A* is the set of true predictors, *B* is the set of variables selected by the method (also called hits), and *k* and *n_B_* are their respective sizes. AltSens measures the average highest absolute correlation value between a true predictor and any variable selected by the method, and AltFDP measures the average highest absolute correlation value between a selected variable and any of the true predictors. AltFDP equals 1 – the average highest absolute correlation value between a selected variable and any of the true predictors. If the set of selected covariates includes all true predictors, these alternative metrics correspond to the classical sensitivity and FDP measures. Given that |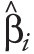 (*X_i_*, *X_j_*)| ≤ 1, AltSens is always greater than the sensitivity, and AltFDP is always smaller than the FDP.

### Extended Variable Selection Protocol

The argument could be made that to increase sensitivity and to avoid missing important signals, one should not look only at the selected exposures but also at all exposures that are highly correlated (i.e., at a level > α, where α varies between 0.6 and 0.9) to these hits. The resulting sensitivity and FDP were computed for this approach.

## Results

### Correlation Structure Used for Generating Exposures

The **Σ** matrix was defined as the nearest positive definite matrix to the INMA correlation structure and only marginally differed from its parent: 75% of the absolute differences were < 0.01, and 95% were < 0.05. The large majority (83%) of absolute correlations between exposures in Σ were < 0.2, but 78% of the exposures were correlated at a level > 0.6 with at least one other exposure (see Figure S1).

### Performance Assessment for Scenario Set 1

**Figure 1:**
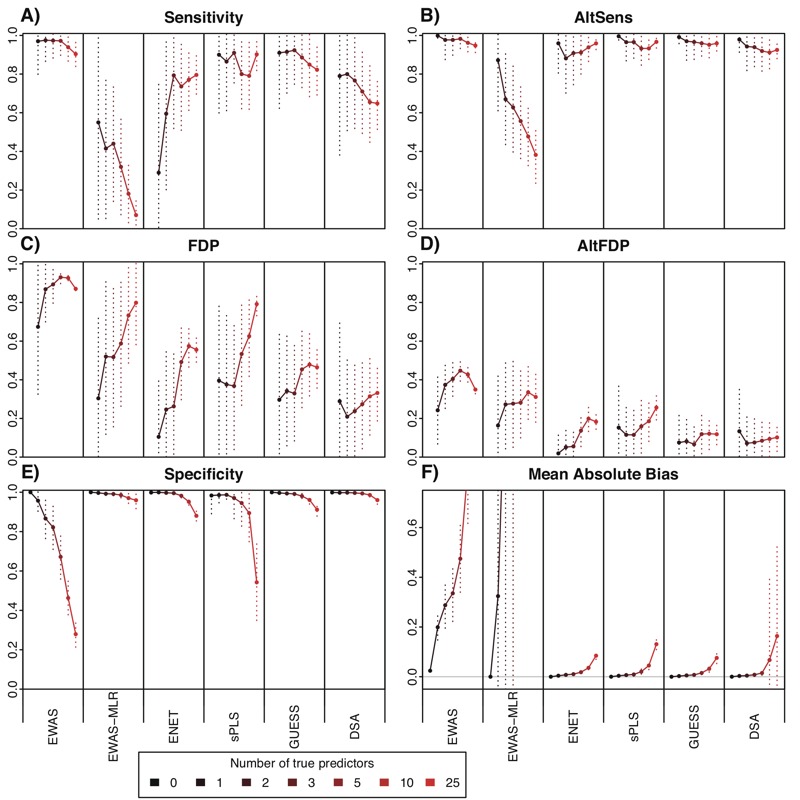
Performances of the statistical methods for scenario set 1. Model performances are summarized by their sensitivity (*A*), alternative sensitivity (AltSens, see "Methods") (*B*), false detection proportion (FDP) (*C*), alternative FDP (AltFDP, see "Methods") (*D*), specificity (*E*) and mean absolute bias (*F*). For each scenario defined by a number of true predictors varying from 0 to 25, statistics over the 100 runs are summarized by their mean (dot), and the variability of each statistic is summarized by 1 standard error in both directions from the average value (vertical dotted line). DSA, Deletion/substitution/addition; ENET, elastic net; EWAS, environment-wide association study; EWAS-MLR, EWAS-multiple linear regression; GUESS, Graphical Unit Evolutionary Stochastic Search; sPLS, sparse partial least squares.

**Figure 2:**
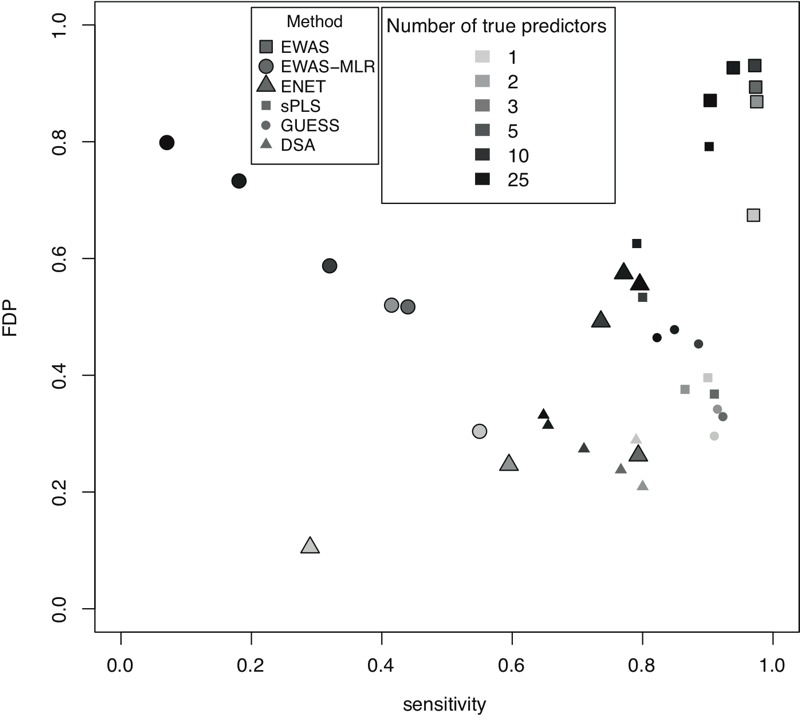
Sensitivity and false discovery proportion (FDP) for scenario set 1. For each scenario defined by a number of true predictors varying from 0 to 25, for each statistical method, sensitivity and FDP over 100 runs are summarized by their mean values. DSA, Deletion/substitution/addition; ENET, elastic net; EWAS, environment-wide association study; EWAS-MLR, EWAS-multiple linear regression; GUESS, Graphical Unit Evolutionary Stochastic Search; sPLS, sparse partial least-squares.

The simulation results for scenario set 1 are presented in Figures 1 and 2 and in Table 1.With true predictors drawn fully at random, the per-scenario average (standard error) absolute pairwise correlation among true predictors ranged between 0.12 and 0.15 (0.12 and 0.16).

**Table 1:** Statistical performances of the statistical methods for scenario set 1. Results are given as mean [min; max] over the seven scenarios, each defined by a different number of true predictors: *k* = 0, 1, 2, 3, 5, 10, and 25 (100 runs per scenario).

Method	Sensitivity	AltSens	FDP	AltFDP	Specificity	n_B_/*k*	Mean absolute bias	Mean absolute bias for TP
EWAS	0.96 [0.90;0.98]	0.97 [0.95;1.00]	0.86 [0.67;0.93]	0.37 [0.24;0.45]	0.72 [0.28;1.00]	11.27 [0.00;16.66]	0.59 [0.02;1.98]	0.04 [0.02;0.10]
EWAS-MLR	0.33 [0.07;0.55]	0.60 [0.38;0.87]	0.58 [0.30;0.80]	0.27 [0.16;0.34]	0.99 [0.96;1.00]	0.86 [0.00;1.33]	9.00 [0.00;42.72]	0.67 [0.45;0.93]
ENET	0.66 [0.29;0.80]	0.93 [0.88;0.96]	0.37 [0.10;0.57]	0.11 [0.02;0.20]	0.97 [0.88;1.00]	1.15 [0.34;1.87]	0.02 [0.00;0.08]	0.74 [0.54;0.98]
sPLS	0.86 [0.79;0.91]	0.96 [0.93;1.00]	0.52 [0.37;0.79]	0.16 [0.12;0.26]	0.90 [0.54;0.99]	3.59 [2.39;4.78]	0.03 [0.00;0.13]	0.46 [0.38;0.57]
GUESS	0.88 [0.82;0.92]	0.97 [0.95;0.99]	0.39 [0.30;0.48]	0.10 [0.07;0.12]	0.98 [0.91;1.00]	1.45 [0.09;1.79]	0.02 [0.00;0.08]	0.37 [0.33;0.42]
DSA	0.73 [0.65;0.80]	0.94 [0.91;0.98]	0.28 [0.21;0.33]	0.09 [0.07;0.13]	0.99 [0.96;1.00]	0.95 [0.26;1.38]	0.04 [0.00;0.16]	0.51 [0.31;0.89]
Abbreviations: AltFDP, alternative definition of the false discovery proportion (see "Methods" for definition); AltSens, alternative definition of the sensitivity (see "Methods" for definition); DSA, deletion/substitution/addition; ENET, elastic net; EWAS, environment-wide association study; EWAS-MLR, EWAS-multiple linear regression; FDP, false discovery proportion; GUESS, Graphical Unit Evolutionary Stochastic Search; n_B_/*k*, number of variables selected by the method (n_B_) divided by the number of true predictors (*k*); sPLS, sparse partial least squares; TP, true predictors.								

Over all investigated numbers of true predictors (i.e., *k* = 0, 1, 2, 3, 5, 10, 25), the EWAS approach yielded a sensitivity > 90% but a specificity as low as 28% and an FDP > 67% (owing to the selection of a large number of exposures as measured by n_B_/*k* in Table 1). The AltFDP ranged between 24% and 45% across simulations. The mean absolute bias was large (range, 0.02 to 1.98), but restricted to the true predictors only, it was the smallest of all the statistical methods (≤ 0.10 vs. ≥ 0.30 for all other methods; see Figure S2).

When EWAS was followed by a multiple linear regression step (EWAS-MLR), the FDP improved over all scenarios (range, 30–80%), as did the specificity (> 95% over all scenarios); however, these improvements came at the cost of a much lower sensitivity (< 56% over all scenarios). The AltSens was between 38% and 87%, and the AltFDP was between 16% and 34%. The mean absolute bias was large (9.00 on average over all scenarios).

The results were similar when using other corrections for multiple testing (see Figure S3). If no adjustment for multiple comparisons was applied, the FDP obtained with this modified EWAS was > 89% and AltFDP was > 42%.

The GUESS, sPLS, ENET and DSA methods all showed lower FDPs than EWAS or EWAS-MLR. On average (5th percentile; 95th percentile) over all scenarios and for these four statistical methods, sensitivity was 78% (60%; 91%), FDP was 39% (21%; 62%), specificity was 96% (89%; 100%), AltSens was 95% (91%; 99%), and AltFDP was 12% (5%; 20%). The mean absolute bias was 0.03 (0.00; 0.11), and it was 0.52 (0.32; 0.89) when restricted to the true predictors only (see Figure S2). On average, these methods selected 1.79 times the number of true predictors (n_B_/*k* in Table 1). On average, DSA and GUESS demonstrated a better compromise between sensitivity and FDR (average values: 81% and 34%, respectively) than sPLS and ENET (average values: 76% and 44%, respectively), with DSA slightly favoring a high sensitivity and GUESS favoring a low FDP (Figure 2). However, none of these statistical methods outperformed the others across all scenarios and indicators investigated.

Over all methods, as the number of true predictors increased, the variable selection performances generally decreased: FDP and AltFDP substantially increased across all statistical methods (on average, +29%, +9% between *k* = 1 and *k* = 25, respectively), sensitivity and AltSens decreased slightly for all methods except EWAS-MLR and ENET (–7% and –4% between *k* = 1 and *k* = 25, respectively), and mean absolute bias increased (particularly for the EWAS-based approaches). Sensitivity and AltSens largely decreased for EWAS-MLR and largely increased for ENET. However, care should be taken in interpreting these trends because an increased number of true predictors is accompanied by an increased signal to noise ratio (*R* ^2^ of the true model), but it is also accompanied by an increased risk that some true predictors are highly correlated.

### Performance Assessment Under Alternative Versions of the Scenarios

Scenarios in which true exposures were selected such that all of their absolute pairwise correlations were < 0.2 (set 2) or > 0.5 (set 3) showed that the higher the level of correlation among the true predictors, the lower the sensitivity for the ENET, GUESS, and DSA methods (and to a lesser extent for the EWAS-MLR method), and the higher the mean absolute bias, mostly for the EWAS-based and DSA approaches (see Figure S4). FDP was affected for the ENET, sPLS, and DSA methods, although not in a consistent direction. Apart from a large AltFDP decrease for the ENET method, the specificity and the alternative definitions of both sensitivity and FDP were mildly affected. It should be noted that selecting predictors with high pairwise correlation yielded an increase in the variance of the error term used in the simulations.

**Figure 3:**
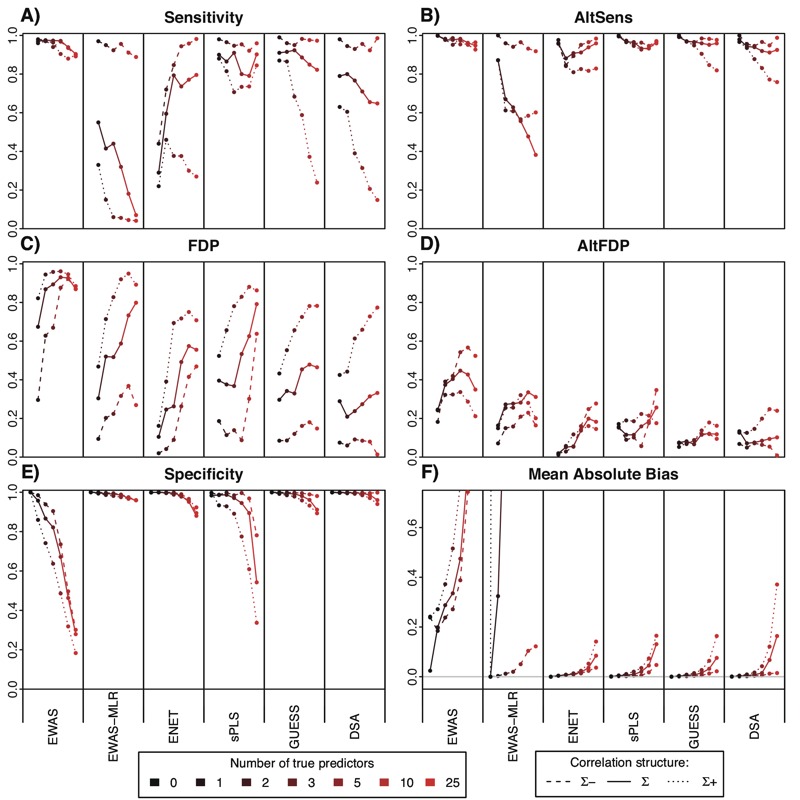
Performances of the statistical methods according to the amount of correlation between the exposures. Model performances are summarized by their sensitivity (*A*), alternative sensitivity (AltSens*,* see "Methods") (*B*), false detection proportion (FDP) (*C*), alternative FDP (AltFDP*,* see "Methods") (*D*), specificity (*E*) and mean absolute bias (*F*). The solid line connects results for exposures generated from a multivariate normal distribution with covariance matrix ∑ (scenario set 1); the dashed line connects results obtained with covariance matrix ∑^–^ (correlations divided by 2 compared with ∑, scenario set 4), and the dotted line connects results obtained with covariance matrix ∑^+^ (correlations multiplied by 2 compared with ∑ and upper bounded by 1, scenario set 5). For each scenario defined by a number of true predictors varying from 0 to 25, statistics over the 100 runs are summarized by their mean (dot). DSA, Deletion/substitution/addition; ENET, elastic net; EWAS, environment-wide association study; EWAS-MLR, EWAS-multiple linear regression; GUESS, Graphical Unit Evolutionary Stochastic Search; sPLS, sparse partial least squares.

Generating exposures from a correlation matrix with higher (scenario set 4) or lower (scenario set 5) levels of correlation (Figure 3) did not alter the methods’ comparison, but it had a major impact on the sensitivity, FDP, and mean absolute bias: the higher the correlation among the exposures, the worse the performances of the methods. For correlation levels divided by 2 compared with scenario set 1, the sensitivity was > 85% for all scenarios and statistical methods (except ENET for *k* < 3), and FDP decreased on average by 23% compared with the same scenario in set 1. The AltSens, the FDP, and the specificity were less sensitive to the overall correlation of exposures and were affected less consistently.

Deviation from the assumption of normally distributed exposures (scenario set 6) led to results analogous to those obtained for scenario set 1, except that the EWAS-MLR method showed better results for the bootstrapped data but did not compete with the other methods (see Figure S5).

Considering varying effect sizes for true predictors (drawn from a uniform distribution in [0.5, 1.5], scenario set 7) did not alter the methods comparison and had a limited impact on the statistical performances: sensitivity and AltSens were moderately reduced (–10% to –7% on average compared with the same scenario in set 1), and specificity, mean absolute bias (except for EWAS-based methods), FDP, and AltFDP were not affected (see Figure S6).

### Extended Variable Selection Protocol

In scenario set 1, when augmenting the list of variables selected by a method with variables that were correlated to any of the hits above some threshold α, a substantial increase in the FDP was observed (except for EWAS-based methods, for which the FDP was already high), even for α as high as 0.8 or 0.9 (see Table S2).

## Discussion

We tested the ability of several established statistical approaches to identify, from a large set of correlated exposures, those causally related to a continuous health outcome. We mostly relied on sensitivity and false detection proportion to assess the statistical methods’ performances: specificity was always high in our simulations (which can be at least partially attributed to our assumption that no more than 25 of the 237 exposure variables were associated with the outcome), making FDP a more discriminating criterion. In addition to the classical measures of sensitivity and FDP, we introduced alternative definitions to account for the fact that false positives that are correlated to a true predictor might actually provide information that can be used to identify this true predictor.

The EWAS-related approaches performed poorly under the scenarios investigated. EWAS captured a large number of (falsepositive) covariates (average FDP of 86% for scenario set 1), irrespective of the procedure used for correcting multiple hypothesis testing (Benjamini and Hochberg, Benjamini and Yekutieli, and permutation-based FDR procedures, or Bonferroni correction). This high FDP was mostly because FDR procedures assume that the statistics (here, the *p*-values) are unbiased, whereas in our simulations, there was a high potential for confounding owing to independently fitting regression models on correlated exposures. However, compared with the other methods investigated, EWAS performed the best in estimating the true predictor coefficient values. When EWAS was followed by a multiple linear regression step (EWAS-MLR), a small proportion of true predictors were captured (average sensitivity of 33% for scenario set 1). However, these two statistical methods still performed much better than if no correction for multiple comparisons was applied, which in the literature corresponds to the association of each exposure with the outcome being considered sequentially in different publications. For these two methods, the AltFDP remained relatively high (32% on average for scenario set 1), suggesting that in the investigated scenarios, many of the variables mistakenly selected by these approaches were not strongly correlated to a true predictor.

With the ENET, sPLS, GUESS, and DSA approaches, most true predictors were selected by the method (average sensitivity of 78% for scenario set 1), and a substantial proportion of exposures were mistakenly suspected to be associated with the outcome (average FDP of 39% for scenario set 1). For these four statistical methods, exposures that were mistakenly selected were on average highly correlated to at least one of the true predictors (average AltFDP of 12% for scenario set 1). Similarly, when a true predictor was not selected by these methods, it was likely that a highly correlated covariate was selected instead (average AltSens of 95% for scenario set 1). None of the multivariate statistical methods tested clearly outperformed the others across all scenarios and properties examined. Globally, DSA and GUESS demonstrated the best compromise between sensitivity and FDP, with DSA favoring high sensitivity and GUESS favoring low FDP. Deviating from the assumption of normally distributed exposures or from the assumption of even effect sizes for true predictors did not alter the methods comparison. However, GUESS and DSA were the most affected by high correlation levels among true predictors (scenario set 3), whereas EWAS and sPLS were less sensitive to this feature. Other factors such as ease of use, ability to force in confounders, and accommodation for different study designs (e.g., longitudinal designs) or for nonlinear exposure–response relationships (e.g., using splines) may also be important when choosing between these methods.

The argument has been made that selecting variables highly correlated to the true predictors should not be considered a false selection per se (Frommlet et al. 2012), and our alternative definitions of FDP and sensitivity were actually developed under this logic. As indicated by the relatively high values of these modified criteria for the four multivariate statistical methods, most of the true predictors are likely to belong to the set of exposures that is highly correlated to the variables selected. Thus, considering the “hits” and their correlated covariates may be a way to capture the true predictors. There are several things to note when considering such an extended variable selection protocol and our findings in general: *a*) in genetic studies, one can identify known and unknown correlated polymorphisms by utilizing the architecture of the genome; this technique may not apply to the exposome because correlations between exposures may arise from a variety of mechanisms (diet, socioeconomic status, etc.), and there is no guarantee that selecting a correlated variable will provide useful information on the causal mechanism linking the true predictors to the outcome. As such, making the distinction between true predictors and predictors correlated to those true predictors is challenging; *b*) lowering the threshold for selection (by including all predictors correlated to a selected predictor) will likely lead to an increased FDP under the usual definition, which may more than offset the benefits (in terms of an increased sensitivity). This effect is exemplified in our results for this protocol, which suggested a substantial increase in the FDP when selecting variables correlated with the hits at a level > 0.8 (see Table S2). In that respect, it is important to stress that our alternative definition of FDP (AltFDP) is not the FDP that would result from the variable selection method induced by AltSens, where predictors highly correlated to the selected ones would also be selected. Instead, it is the FDP that would result from using the original selection protocol, but counting correlated variables as “true predictors,” with a weight proportional to their correlation with the true predictor.

Our simulation work extends that of Sun et al. (2013) to a more realistic context for the exposome in terms of number of exposures and of their correlation structure. We showed that the correlation structure under which the exposures are generated greatly affects the performances of the statistical methods (Figure 3), meaning that the results from Sun et al. and those of any simulation study with fixed correlation structure cannot be generalized in a straightforward way to the exposome context.

Our study relied on several modeling assumptions that must be taken into consideration when discussing the generalizability of our results. First, we assumed no effect measure modification of a covariate on the health outcome by any other covariate (departure from additivity), a situation which may in practice not be true. Incorporating interaction terms would strongly increase the size of the modeling space (e.g., in the present study, 27,966 first-order interactions) and would require extending our statistical methods to test for interactions, using dedicated techniques from all families investigated here (e.g., Li and Zhang 2010). Withdrawing the restriction of binary effect sizes and incorporating varying effect sizes in the simulation did not alter the FDP, and it only artificially reduced the statistical power to detect weaker effects (reduction in sensitivity of 10% on average). These results can be explained by a ceiling effect; that is, the already high sensitivity could not be improved for exposures with higher than average effects to the same extent as it could be reduced for exposures with lower than average effects. Overall, the induced sensitivity loss was consistent across all methods and did not help in further discriminating the statistical methods under investigation. Importantly, we did not consider measurement error or misclassification in exposure covariates, although they have a potentially large impact on statistical power and bias, particularly in the case of classical type error (de Klerk et al. 1989; Perrier et al. 2016; Rappaport et al. 1995). As a result, method performance may be hampered in real-life situations, but there is no *a priori* reason to think that the statistical methods investigated in this study would be differentially affected by these issues. We further assumed that exposures were normally distributed. Deviating from this assumption did not alter the performances of the methods. Finally, similarly to Sun et al. (2013), we used a limited set of statistical methods that all borrowed from the linear regression framework. Alternative approaches such as profile regression, cluster analysis, or other machine learning methods could complement this portfolio of approaches but could not be straightforwardly compared with our set of regression-based approaches.

## Conclusions

Relying on a realistic exposome structure, we screened a large set of correlated exposures, of which only a small number were directly associated with a continuous outcome. Our results suggest that the multivariate methods investigated are preferable to univariate approaches to investigating the exposome: despite not achieving a low FDP, they showed satisfactory statistical performance and represented different balances between sensitivity and FDP. Based on our performance metrics, we identified DSA and GUESS as providing somewhat better performance, but this was not true across all scenarios and properties examined, and in real case analyses, methodological choices should also be guided by computational complexity and flexibility considerations such as the ability to accommodate confounders. The performances of the statistical methods were strongly influenced by the correlation among the exposome covariates, illustrating an issue inherent to exposome research, namely that the statistical methods investigated could not efficiently differentiate between true predictors and correlated covariates.

**Editor’s note:** The authors note that to better summarize the results for scenario set 1, Table 1 has been revised to show mean (minimum, maximum) values of each statistic when averaged over the seven scenarios, in place of the mean (5th, 95th percentile) values over all simulation runs for all seven scenarios (100 runs per scenario) in scenario set 1.

## Supplemental Material

(1.1 MB) PDF

(502 KB) ZIP
