# Supplementary material for: A Systematic Comparison of Linear Regression–Based Statistical Methods to Assess Exposome-Health Associations
Source: Environ Health Perspect. 2016 May 24;124(12):1848–56. doi: 10.1289/EHP172 (PMC5132632; doi:10.1289/EHP172)
Supplement: (1.1 MB) PDF [file EHP172.s001.acco.pdf]

Environ Health Perspect
DOI: 10.1289/EHP172

Note to readers with disabilities: *EHP* strives to ensure that all journal content is accessible to all readers. However, some figures and Supplemental Material published in *EHP* articles may not conform to 508 standards due to the complexity of the information being presented. If you need assistance accessing journal content, please contact ehp508@niehs.nih.gov. Our staff will work with you to assess and meet your accessibility needs within 3 working days.

Supplemental Material

A Systematic Comparison of Linear Regression-Based Statistical Methods to Assess Exposome-Health Associations

Lydiane Agier, Lützen Portengen, Marc Chadeau-Hyam, Xavier Basagaña, Lise Giorgis-Allemand, Valérie Siroux, Oliver Robinson, Jelle Vlaanderen, Juan R. González, Mark J. Nieuwenhuijsen, Paolo Vineis, Martine Vrijheid, Rémy Slama, and Roel Vermeulen

Table of Contents

Figure S1. Correlation among exposure covariates presented as the heatmap (A) and histogram of absolute values (B) of all Pearson pairwise correlation coefficients in Σ (the semi-definite matrix the closest to the correlation matrix derived from the INMA cohort), and as the highest absolute correlation per covariate (C).

Supplemental Material S1

Setting the residual variance for the simulations

Expected FDP in the presence of strong correlations

Supplemental Material S2

Functions

Generating the Data

Applying the Regression-Based Methods

Excel File Table S1. See "Additional Files" below

Table S2. Sensitivity and FDP obtained when augmenting the list of variables selected by a method with variables that are correlated (in absolute value) to those variables above some threshold α (with N an average number of variables in total), with α values varying in the range 0.6,0.7,0.8,0.9,1 (1 standing for the scenarios set 1). The table also includes results for

the hypothetical situation in which we had a perfect model (oracle) to illustrate the limitations of having highly correlated data. Results are given for scenarios with k=0,1,2,3,5,10,25 true predictors.

Figure S2. Additional parameters characterizing the performances of the statistical methods for scenarios set 1. Model performances are summarized by n_B the number of variables selected by the method (A), the ratio n_B/k with k the number of true predictors (=n_B when $k = 0$) (B), the error variance σ^2 (C), the mean bias (all coefficient values being equal to 1) (D), the mean absolute bias computed over the true predictors (E) and over the other variables that are not true predictors (F). For each scenario defined by a number of true predictors varying from 0 to 25, statistics over the 100 runs are summarized by their mean (dot) and the variability of each statistics is summarized using the one standard error both ways from the average value (vertical dotted line). DSA: Deletion/substitution/addition; ENET: Elastic net; EWAS: Environment-wide association study; EWAS-MLR: EWAS-Multiple Linear Regression; GUESS: Graphical Unit Evolutionary Stochastic Search; sPLS: Sparse partial least-squares; TP: True Predictors.

Figure S3. Performances of the statistical methods for scenarios set 1 using alternative multiple hypothesis testing corrections for the EWAS and EWAS-MLR methods: the Bonferroni (Bon), permutation (perm) and Benjamini and Hochberg (BH) corrections. For each scenario defined by a number of true predictors varying from 0 to 25, statistics over the 100 runs are summarized by their mean (dot) and the variability of each statistics is summarized using the one standard error both ways from the average value (vertical dotted line). EWAS: Environment-wide association study; EWAS-MLR: EWAS-Multiple Linear Regression.

Figure S4. Performances of the statistical methods according to the amount of correlation between the true predictors. The full line connects the results for scenarios with correlations between the true predictors (in absolute values) in the [0,1] range (scenarios set 1); the dashed line in the [0,0.2] range (scenarios set 2) and the dotted line in the [0.5,1] range (scenarios set 3). For each scenario defined by a number of true predictors varying from 0 to 25 (to 10 for set 3, Σ not containing 25 exposures that are correlated at a level >0.5), statistics over the 100 runs are summarized by their mean (dot). DSA: Deletion/substitution/addition; ENET: Elastic net; EWAS: Environment-wide association study; EWAS-MLR: EWAS-Multiple Linear Regression; GUESS: Graphical Unit Evolutionary Stochastic Search; sPLS: Sparse partial least-squares.

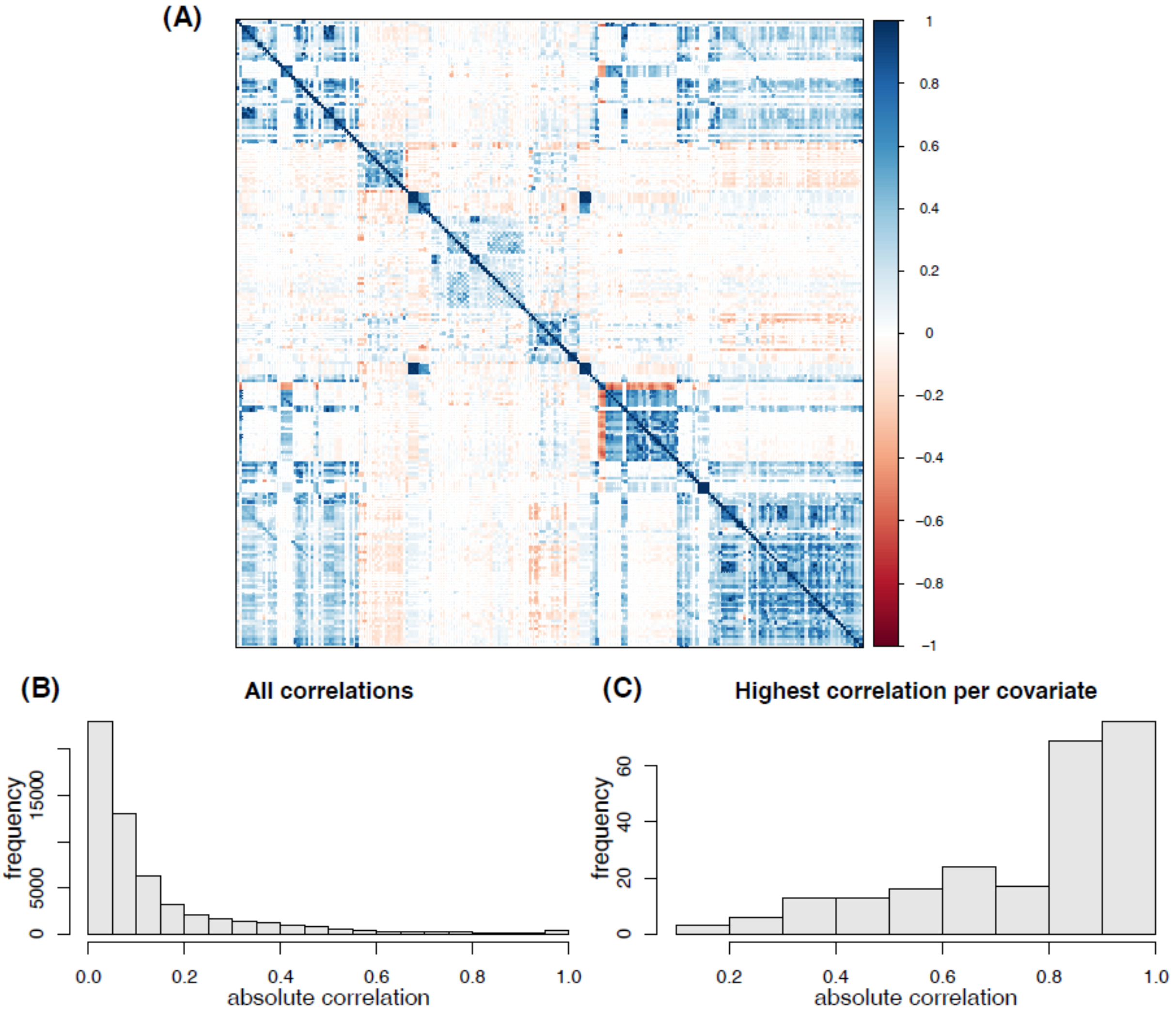

Figure S1. Correlation among exposure covariates presented as the heatmap (A) and histogram of absolute values (B) of all Pearson pairwise correlation coefficients in Σ (the semi-definite matrix the closest to the correlation matrix derived from the INMA cohort), and as the highest absolute correlation per covariate (C).

Figure S5. Performances of the statistical methods when deviating from the assumption of normally distributed exposures. The full line connects the results for scenarios set 1 (exposures generated from a normal distribution); the dotted line corresponds to the same scenarios being tested on exposure data bootstrapped from the INMA environmental data (scenarios set 6). For each scenario defined by a number of true predictors varying from 0 to 25, statistics over the 100 runs are summarized by their mean (dot). DSA: Deletion/substitution/addition; ENET: Elastic net; EWAS: Environment-wide association study; EWAS-MLR: EWAS-Multiple Linear Regression; GUESS: Graphical Unit Evolutionary Stochastic Search; sPLS: Sparse partial least-squares.

Figure S6. Performances of the statistical methods for varying effect sizes among true predictors. The full line connects the results for scenarios set 1 (all effect sizes equal to 1); the dotted line corresponds to the same scenarios except with effect sizes generated from a uniform distribution in [0.5,1.5] (scenarios set 7). For each scenario defined by a number of true predictors varying from 0 to 25, statistics over the 100 runs are summarized by their mean (dot). DSA: Deletion/substitution/addition; ENET: Elastic net; EWAS: Environment-wide association study; EWAS-MLR: EWAS-Multiple Linear Regression; GUESS: Graphical Unit Evolutionary Stochastic Search; sPLS: Sparse partial least-squares.

Additional Files

Supplemental Code and Data Zip File

Supplemental Code and Data Zip File Index

Excel File Table S1: Matrix of all Pearson pairwise correlation coefficients in Σ, the semi-definite matrix the closest to the correlation matrix derived from the INMA cohort

Supplemental Material S1

Setting the residual variance for the simulations

Consider the function used to generate the health outcome:

$$Y = \sum_{i=1}^{237} \beta_i X_i + \epsilon\,, \epsilon \sim N(0, \sigma^2)$$

and the set A of true predictors ($i \in A$: $\beta_i=1$; $i \notin A$: $\beta_i=0$). If all predictors are orthogonal and there is only one true predictor (i.e. the size of A = 1), the power of a study with N=1200 observations is fully determined by the residual variance. Under these circumstances the expected standard error of the beta coefficients is directly proportional to the square root of the residual variance of the model (and the t-statistic inversely proportional). If the number of true predictors grows larger, the estimated residual standard error will be equal to $\sqrt{\sigma^2}$ (on average) only for models in which all true predictors are included in the model, and will be larger for models that include only one variable at a time (e.g. those used for the EWAS approach).

We are interested in the *relative* performance of the selection methods, and the power of the simulated studies is not of interest in itself. The choices for specific values for β and σ^2 do affect the (absolute) levels of sensitivity, specificity, etc… that the methods can attain, and we therefore choose them in such a way that we increase our chances of picking up differences between methods (f.i. we try to avoid situations in which all methods have close to 100% (or 0%) sensitivity). It is impossible to fix the sensitivity across scenarios with an increasing number of true predictors for all the regression approaches simultaneously, and we therefore chose to fix the error variance in a way that keeps the total variance that can be explained by the set of true predictors (or equivalently, the signal to noise ratio) to be constant across simulations within a scenario and proportional to the number of true predictors in a scenario. For the scenario with only one true predictor, the error variance was set to 32.33, which results in an overall R^2 of 3%. By reducing the error variance by (#true predictors-1) for scenarios with more than one true predictor we allow R^2 to increase linearly with the #true predictors as *#true predictors**3%.

For non-orthogonal designs setting R^2 becomes more complicated. The expected maximum proportion of variance in Y than can be explained by our models is a function of the variance in the linear predictor and the error variance, as follows:

$$\widehat{\max(R^2)} = var(\sum_{i=1}^{237} \beta_i X_i) / (var(\sum_{i=1}^{237} \beta_i X_i) + var(\epsilon))$$

If the true predictors are correlated, the variance of the linear predictor is larger than the sum of the variances of the individual predictor variables. The maximum R^2 (or signal to noise ratio) will therefore be different between simulations within a scenario. By allowing the error variance to depend on the variance of the linear predictor instead of the number of true predictors, we can still fix R^2:

$\sigma^2 = var(\sum_{i=1}^{237} \beta_i X_i) \times \frac{R^2}{1-R^2}$, with R^2 = *#true predictors* × 3%

Expected FDP in the presence of strong correlations

Most of the correlations in the matrix used to generate the exposome in our simulations are quite low (absolute r_p<0.1 for more than 64% of the entries), but there is a small proportion (0.7%) of predictors that are quite strongly correlated (r_p>0.8) . As no attempt was made to pre-filter or collate highly correlated predictor variables (because that seems at odds with the exposome concept), there is a considerable probability that one of the true predictors is strongly correlated with one of the variables that do not belong to this set. Although we have tried to take account of this by introducing our alternative measure of FDP, it is still of interest to investigate what level of FDP should be expected under these circumstances. We therefore calculated, for each simulation, the FDP that would result from a selection method that selected all the true predictors (a so-called oracle procedure) augmented by all the variables that were correlated to at least one of the true predictors above a threshold value (αr).

The argument could also be made that in order to increase sensitivity and avoid missing important signals, one should not look only at the selected exposures, but also consider all exposures highly correlated to these selected ones. This is similar to the approach described above, but with the initial set of variables selected using one of the methods investigated in this study instead of an oracle procedure (see Supplemental Material, Excel File Table S1).

Supplemental Material S2

```
################################################################################
##                                          FUNCTIONS
################################################################################


################################### simResponseReg()
simResponseReg <-
function(Exp,Nexp=NA,beta=NA,betaVar=NA,sigmaE=NA,sigmaY=NA,R2=NA,CorrLim=
c(0.1,0.4),reduced=FALSE){
     if(any(is.null(colnames(Exp)))) stop("Exp should have columns names")
          beta2 <- beta; betaVar2 <- betaVar ; Nexp2 <- 0
          if(is.list(beta2)){
             Nexp2 <- length(beta2)
             beta <- beta[[1]]
             while(all(is.na(beta2[length(beta2)]))) beta2 <- beta2[-length(beta2)]
          }
          if(is.list(betaVar2)){
              Nexp2 <- max(length(betaVar2),Nexp2)
              betaVar <- betaVar[[1]]
              while(all(is.na(betaVar2[length(betaVar2)]))) betaVar2 <- betaVar2[-
length(betaVar2)]
          }
          if(length(CorrLim)!=2 | any(CorrLim>1)| any(CorrLim<0)) print("CorrLim must be a
vector of length 2")
          if(all(is.na(c(Nexp,beta2,betaVar2)))) stop("you need to specify the number of
exposure variables to consider (through Nexp or beta and betaVar)")
          if(is.na(Nexp)){
               if(!is.matrix(beta) & !is.matrix(betaVar)) Nexp <-
max(length(betaVar),length(beta),Nexp2)
               if(is.matrix(beta) | is.matrix(betaVar)) Nexp <-
max(ncol(betaVar),ncol(beta),Nexp2)
          }
          if(length(sigmaE)>1 | length(R2)>1 | length(sigmaY)>1) stop("sigmaE, sigmaY and
R2 should not be of length greater than 1")
          if(is.na(sigmaE)+is.na(sigmaY)+is.na(R2)==3){
               R2 <- 0.1
               print("sigmaE,sigmaY and R2 not specified: R2 set to 10%")
          }
          if(is.na(sigmaE)+is.na(sigmaY)+is.na(R2)!=2) stop("only one of sigmaE,sigmaY or
R2 should be specified")
          # defines whether we are considering interactions and simulating beta
          interaction <- var <- TRUE
          if(is.list(beta2)) if(length(beta2)==1) beta2 <- unlist(beta2)
          if(is.list(betaVar2)) if(length(betaVar2)==1) betaVar2 <- unlist(betaVar2)
          if(!is.list(beta2) & !is.list(betaVar2)){
               if(all(is.na(betaVar) | betaVar==0)) var <- FALSE
               if(!is.matrix(beta) & !is.matrix(betaVar)) interaction <- FALSE
               if(is.matrix(beta)){
```

```
            betaNoDiag <- c(beta[upper.tri(beta)],beta[lower.tri(beta)])
            betaVarNoDiag <- c(betaVar[upper.tri(betaVar)],beta[lower.tri(betaVar)])
            if(all(is.na(betaNoDiag))) interaction <- FALSE
            if(!is.matrix(betaVar) & all(betaNoDiag==0 | is.na(betaNoDiag))) interaction <-
FALSE
            if(is.matrix(betaVar)) if(all(betaNoDiag==0 | is.na(betaNoDiag)) &
all(betaVarNoDiag==0)) interaction <- FALSE
          }
        }
        if(is.list(beta2)) if(all(is.na(unlist(betaVar)) | unlist(betaVar)==0)) var <- FALSE
        # corrects the size of beta
        if(interaction==TRUE){
          if(Nexp2<2){
            if(!is.matrix(beta)) stop("beta should be a matrix")
            if(!all(dim(beta)==Nexp)) stop("beta should be a squared matrix or same size as
betaVar")
            if(all(is.na(beta[upper.tri(beta)]))) beta <- t(beta)
            if(isSymmetric(beta) | all(is.na(beta[lower.tri(beta)]))) beta <-
c(diag(beta),beta[upper.tri(beta)])
            if(is.matrix(beta)) stop("beta should be symmetric or triangular")
            if(var==TRUE){
             if(!is.matrix(betaVar)) stop("betaVar should be a matrix")
             if(!all(dim(betaVar)==Nexp)) stop("betaVar should be a squared matrix of same
size as beta")
             if(all(is.na(betaVar[upper.tri(betaVar)]))) betaVar <- t(betaVar)
             if(isSymmetric(betaVar) | all(is.na(betaVar[lower.tri(betaVar)]))) betaVar <-
c(diag(betaVar),betaVar[upper.tri(betaVar)])
             if(is.matrix(betaVar)) stop("betaVar should be symmetric")
          }}
          if(Nexp2>=2){
            beta <- betaVar <- array(NA,0)
            for(i in 1:max(length(beta2),length(betaVar2))){
               if(length(beta2)<i) beta2[[i]] <- 0
               if(length(betaVar2)<i) betaVar2[[i]] <- 0
               leng <-  ncol(combn(Nexp,i))
               if(i==1) leng <- Nexp
               if(length(beta2[[i]])==1) beta <- c(beta,rep(beta2[[i]],leng))
               if(length(beta2[[i]])==leng & leng!=1) beta <- c(beta,beta2[[i]])
               if(length(betaVar2[[i]])==1) betaVar <- c(betaVar,rep(betaVar2[[i]],leng))
               if(length(betaVar2[[i]])==leng & leng!=1) betaVar <- c(betaVar,betaVar2[[i]])
        }}}
        if(interaction==FALSE){
            if(all(is.na(beta))) beta <- 1
            if(length(beta)==1) beta <- rep(beta,Nexp)
            if(is.matrix(beta)) beta <- diag(beta)
            if(var==TRUE){
               if(length(betaVar)==1) betaVar <- rep(betaVar,Nexp)
               if(is.matrix(betaVar)) betaVar <- diag(betaVar)
            }
```

```
            if(length(Nexp)!=1 | length(beta)!=Nexp) stop("Nexp and beta are not of consistent
size")
            if(var==TRUE & length(betaVar)!=Nexp) stop("Nexp and betaVar are not of
consistent size")
        }
        # defining the empirical correlation matrix
        Mat <- abs(cor(Exp))
        diag(Mat) <- NA
        wh <- colnames(Mat)
        # defining a set of exposome variables with the expected correlation values
        if(Nexp>1){
           wh <- submatFindSimpl(Exp,range=CorrLim,Nvar=Nexp)
             corT <-  cor(Exp[,which(colnames(Exp)%in%wh)])
            diag(corT) <- NA
            if(min(abs(corT),na.rm=T)<CorrLim[1] | max(abs(corT),na.rm=T)>CorrLim[2])
stop("error in accounting for CorrLim")
        }
          if(Nexp>ncol(Exp)) stop("Exp does not contain enough exposure variables with the
required correlation")
        wh <- sample(wh, size=Nexp)
        CovMat <- as.matrix(Exp[,wh])
        colnames(CovMat) <- wh
        # adding interaction if requested
        if(Nexp>1 & interaction==TRUE)
              CovMat <- cbind(CovMat,AddInteract(CovMat,max=max(2,Nexp2)))
        wh <- which(is.na(beta))
        if(length(wh)>0){
            CovMat <- matrix(CovMat[,-wh],ncol=ncol(CovMat)-
length(wh),dimnames=list(1:nrow(CovMat),colnames(CovMat)[-wh]))
            beta <- beta[-wh]
            if(var==TRUE){
             if(!all(is.na(betaVar[wh])|betaVar[wh]==0)) stop("beta should not contain NAs if
betaVar is not NA or 0")
             betaVar <- betaVar[-wh]
             if(any(is.na(betaVar))) betaVar[which(is.na(betaVar))] <- 0
         }}
        # simulating beta values
        if(var==TRUE) beta <- mvrnorm(1,beta,diag(betaVar))
        # computing the response
        mean <- CovMat %*% matrix(beta,ncol=1)
        if(reduced==TRUE) mean <- mean/sd(mean)
        if(!is.na(R2)){
           sigmaE <- var(mean)*(1/R2-1)
           if(R2==0) sigmaE <- 100}
        if(!is.na(sigmaY)) sigmaE <- sigmaY-var(mean)
        if(sigmaE<0) stop("var(E)<0")
        resp <- as.matrix(mean +rnorm(length(mean),0,sqrt(sigmaE)), ncol=1)
        beta <- c(0,beta)
        names(beta) <- c("(Intercept)",colnames(CovMat))
        R2 <- var(mean)/var(resp[,1])
```

```
	return (list(resp=resp, beta=beta,sigmaE=sigmaE,R2=R2))
}

#################################### submatFindSimpl()
submatFindSimpl <- function(Mat,range=c(0,1),Nvar){
	# verifying formats and values of inputs
	if(Nvar>ncol(Mat)) stop("No matrix of the correct size meeting the range criterion")
	if(Nvar <2) stop("Nvar must be at least 2")
	# computing the correlation matrix
	Mat <- abs(cor(Mat))
	diag(Mat)<- NA
	# removing rows with no correlation value in the given range
	wh <- which(apply(Mat,1,min,na.rm=T)>range[2] |
	  apply(Mat,1,max,na.rm=T)<range[1])
	if(length(wh)>0) Mat <- Mat[-wh,-wh]
	# iteratively selecting and testing samples for the correct correlation
	Res <- NA
	samp1 <- sample(1:ncol(Mat), size=ncol(Mat))
	t1 <- 1
	while(t1<=ncol(Mat) & all(is.na(Res))){
	  var <- array(NA,0)
	  t2 <- samp1[t1]
	  while(all(is.na(Res)) & length(t2)>0){
	   if(length(t2)==1) var <- c(var,t2)
	   if(length(t2)>1) var <- c(var,sample(t2, size=1))
	   t2 <- (1:ncol(Mat))[-var]
	   if(length(t2)>1) t2 <- t2[which(apply(as.matrix(Mat[t2,var]<=range[2] &
Mat[t2,var]>=range[1] & Mat[t2,var]<1),1,min)==1)]
	   if(length(t2)==1)
if(min(c(Mat[t2,var]<=range[2],Mat[t2,var]>=range[1],Mat[t2,var]<1))!=1) t2 <- NA
	   if(length(var)==Nvar) Res <- var
	  }
	  t1 <- t1+1
	}
	if(all(is.na(Res))) stop("Not enough variable with the given correlation range")
	return(colnames(Mat)[Res])
}

#################################### EWAS()
EWAS <- function(resp,Exp,beta,interaction=FALSE,add=FALSE){
	require(parallel)

	# build the name for interactions of variables in name
	buildName<- function(name){
		if(length(name)>1) for(i in 2:length(name))
		  name[1] <- paste(name[1],":",name[i],sep="")
		return(name[1])
	}

	if(interaction==1) interaction <- FALSE
```

```
	beta <- cbind(var=gsub("[:]","*",names(beta)),val=beta)
	# verifying formats and values of inputs and computing interactions
	if(interaction==FALSE) interaction <- 0
	if(!all(is.null(colnames(Exp)))) Exp <- Exp[,order(colnames(Exp))]
	# EWAS
	if(interaction!=FALSE) if(interaction>1)
		Exp <- cbind(Exp,AddInteract(Exp,max=interaction))
	p.values <- mclapply(1:ncol(Exp), function(x,Exp){
c(colnames(Exp)[x],summary(lm("resp~var",
data=data.frame(cbind(var=Exp[,x],resp=Exp["resp"]))))$coefficients[2,])},
Exp=cbind(Exp,resp=resp))

	p.values <- cbind(matrix(unlist(p.values), ncol = 5, byrow = TRUE)[,-4])
	colnames(p.values) <- c("var","Est","Sd","pVal")
	p.values <- merge(beta,p.values,by="var",all=TRUE)
	p.values <- p.values[-which(p.values$var%in%c("(Intercept)","Intercept")),]
	return(p.values)
}

################################### testReg()

testReg <- function(Exp,outc, p.values,intTest=1,corr="Bon",parTest=NA,Nperm=NA,
lambda=NA, interaction=NA,EWASonly=FALSE){

  p.values <- p.values[order(as.character(p.values$var)),]
  pVal <- as.numeric(as.character(p.values$pVal))
  Exp <- Exp[,order(colnames(Exp))]
  if(!all(p.values$var==colnames(Exp))) stop("issue in ordering")
  resp <- outc$resp

	# fitting multiple linear model
  if(corr=="Bon") wh <- which(pVal<=0.05/nrow(p.values))
  if(corr=="BH") wh <- which(p.adjust(pVal,"BH")<=0.05)
  if(corr=="BY") wh <- which(p.adjust(pVal,"BY")<=0.05)
  if(!corr%in%c("Bon","BH","BY","")) stop("Please specify a known correction method for
multiple testing")

# EWAS
  wh <- p.values$var[wh]
  p.val <-
data.frame(cbind(var=as.character(p.values$var),EWAS=as.numeric(as.character(p.values$Es
t)),EWAS.TP=as.numeric(p.values[,1]%in%wh)))
  if(EWASonly==TRUE){ RMSE=NA; R2=NA }
# EWAS-LM
  if(EWASonly==FALSE){
   formula <- "~1"
   if(length(wh)>0) for(i in 1:length(wh))
		formula <- paste(formula,"+",wh[i])
   modelLM <- lm(as.formula(paste("resp",formula)),data=cbind(Exp,resp=resp))
   predLM <- predict(modelLM, Exp, se.fit = FALSE)
```

```
  modelLM <-
cbind(var=as.character(names(modelLM$coef)),EWAS_LM=modelLM$coef,EWAS_LM.TP
=as.numeric(summary(modelLM)$coef[,4]<.05))
   p.val <- merge(p.val,matrix(modelLM[-
1,],ncol=3,dimnames=list(NULL,colnames(modelLM))),by="var",all=TRUE)

   R2 <- c(NA,NA,var(predLM)/var(outc$resp))
   RMSE <- c(NA,NA,sqrt(mean((predLM-outc$resp)^2)))
  }

  if(nrow(p.val)!=ncol(Exp)) print("erreur de dim")
  if(!all(p.val$var==colnames(Exp))) print("erreur d'ordre de variables")
  if(any(is.na(p.val[,2])| p.val[,2]=="NaN")) print("erreur pour EWAS")
  names <- p.val[,1]
  p.val <- t(p.val[,-1])
  colnames(p.val) <- names
  p.val <- cbind(RMSE=RMSE,R2=R2,p.val)
  return(p.val)
}

################################### DSAreg()
## to apply the DSA() R function and compute and return the related coefficients
DSAreg <- function(Exp,resp, family = gaussian,maxsize = 15, maxsumofpow = 2,
maxorderint = 2){
        Exp <- data.frame(cbind(data.frame(Exp), resp=resp))
        res <- DSA(resp ~ 1, data = Exp,  family = family, maxsize = maxsize, maxsumofpow
= maxsumofpow, maxorderint = maxorderint ,nsplits=1,usersplits = NULL)
        form <- gsub("I[(]","",colnames(coefficients(res)))
        form <- gsub("[*]",":",gsub("[)]","",gsub("[:^:]1","",form)))
        if(length(grep(":",form))>0){
          nam <- strsplit(form[grep("[:]",form)],":")
          for(j in 1:length(nam)){
            nam[[j]] <- gsub("[[:space:]]","",nam[[j]])
            name <- nam[[j]][1]
            for(k in 2:length(nam[[j]]))
                  name <- paste(name,":",nam[[j]][k],sep="")
            Exp <- cbind(Exp,name=apply(Exp[,nam[[j]]],1,prod))
         }}
         form2 <- "resp~1"
         if(length(form)>1)for(i in 2:length(form)) form2 <- paste(form2,"+",form[i])
         res2 <- lm(form2, data=data.frame(Exp))
         pred <- predict(res2,Exp)
         coef <- summary(res2)$coefficients
         coef <- cbind(var=rownames(coef),valDSAEst=coef[,1])
         return(list(coef=coef, pred=pred))
}

################################### EvaluateModel.cv.glmnet()
EvaluateModel.cv.glmnet <- function(x,y,nfolds=10) {
  n <- nrow(x)
```

```
  folds <- rep(1:nfolds,length.out=n)[sample(n,n)]
  #step 1: do all crossvalidations for each alpha in range 0.5 - 1.0
  alphasOfInterest <- seq(0.5,1,0.1)
  cvs <- lapply(alphasOfInterest,
function(curAlpha){cv.glmnet(x,y,alpha=curAlpha,family="gaussian",nfolds=nfolds,foldid=f
olds,standardize=FALSE)})
  #step 2: collect the optimum lambda for each alpha
  optimumPerAlpha <- sapply(seq_along(alphasOfInterest), function(curi){
    curcvs <- cvs[[curi]]
    curAlpha <- alphasOfInterest[curi]
    indOfMin <- match(curcvs$lambda.min, curcvs$lambda)

return(c(lam=curcvs$lambda.min,alph=curAlpha,cvm=curcvs$cvm[indOfMin],cvup=curcvs$
cvup[indOfMin]))
  })
  #step 3: find the overall optimum
  posOfOptimum <- which.min(optimumPerAlpha["cvm",])
  overall.alpha.min <- optimumPerAlpha["alph",posOfOptimum]
  overall.lambda.min <- optimumPerAlpha["lam",posOfOptimum]

  overall.criterionthreshold <- optimumPerAlpha["cvup",posOfOptimum]
  #step 4: now check for each alpha which lambda is the best within the threshold
  corrected1se <- sapply(seq_along(alphasOfInterest), function(curi){
    curcvs <- cvs[[curi]]
    lams <- curcvs$lambda
    lams[lams<overall.lambda.min] <- NA
    lams[curcvs$cvm > overall.criterionthreshold] <- NA
    lam1se <- max(lams, na.rm=TRUE)
    c(lam=lam1se, alph=alphasOfInterest[curi])
  })
  #step 5: find the best (lowest) of these lambdas
  overall.alpha.1se <- max(corrected1se["alph",is.finite(corrected1se["lam",])])
  overall.lambda.1se <- corrected1se["lam",match(overall.alpha.1se, alphasOfInterest)]

  model <-
glmnet(x=x,y=y,alpha=overall.alpha.min,lambda=overall.lambda.min,family="gaussian",stan
dardize=FALSE)
  coef <- as.matrix(predict(model,type="coefficients",s=as.numeric(overall.lambda.min)))[-1]
  names(coef) <- paste0("C",1:length(coef))
  results.min <- cbind(data.frame(model="ENET",type="MIN"),t(coef))

  model <-
glmnet(x=x,y=y,alpha=overall.alpha.1se,lambda=overall.lambda.1se,family="gaussian",stand
ardize=FALSE)
  coef <- as.matrix(predict(model,type="coefficients",s=as.numeric(overall.lambda.1se)))[-1]
  names(coef) <- paste0("C",1:length(coef))
  results.opt <- cbind(data.frame(model="ENET",type="OPT"),t(coef))

  return(rbind(results.min,results.opt))
}
```

```
#################################### EvaluateModel.cv.spls()
EvaluateModel.cv.spls <- function(x,y,max.K=5,nfolds=10) {
  m <- spls::cv.spls(x=x,y=y,K=1:max.K,eta=c(1e-6,seq(0.1,0.9,0.1),1-1e-
6),fold=nfolds,plot.it=FALSE,scale.x=FALSE,scale.y=FALSE)
  RMSE_H0 <- mean((y-mean(y))^2)
  K.min <- m$K.opt
  eta.min <- m$eta.opt
  if (RMSE_H0<=min(m$mspemat)) {
    K.min <- 0
  }
  if (K.min>0) {
    model <- spls::spls(x=x,y=y,K=K.min,eta=eta.min)
    coef <- as.numeric(coef(model))
  } else {
    coef <- rep(0,ncol(x))
  }
  names(coef) <- paste0("C",1:length(coef))
  results <- cbind(data.frame(model="SPLS",type="MIN"),t(coef))
  return(results)
}

################################## EvaluateModel.R2GUESS()
EvaluateModel.R2GUESS <- function(x,y,MPPI,cutoff=0.02475121) {
  coef <- rep(0,length(MPPI))
  selected.inx <- which(MPPI>cutoff)
  if (length(selected.inx)>0) {
    if (length(selected.inx)>1) {
      model <- cv.glmnet(y=y,x=x[,selected.inx,drop=FALSE],alpha=0)
      coef[selected.inx] <-
as.numeric(predict(model,newx=x[,selected.inx,drop=FALSE],s="lambda.min",type="coef")[
-1])
    } else {
      model <- lm(y ~ x[,selected.inx])
      coef[selected.inx] <- as.numeric(summary(model)$coefficients[-1,1])
    }
  }
  names(coef) <- paste0("C",1:length(coef))
  cbind(data.frame(model="R2GUESS",type="MIN"),t(coef))
}

###############################################################################
##                              GENERATING THE DATA
###############################################################################

library(mvtnorm)

# parameters for generating the correlation matrix X
cormat <- as.matrix(read.csv("cormat.csv")[,-1]) # loading the correlation structure for the
exposome
```

```
N= 1200 # number of individuals to simulate the exposome for

# parameters for generating Y
R2 <- 0.03 # the proportion of variance explained by a unique parameter
NPred <-3 # number of true predictors
corr <- c(0.5,1) # range for absolute correlation amongst true predictors
betaMean <- rep(1, NPred)  # effect sizes values

### generating X
data.X <- data.frame(matrix(rmvnorm(N,rep(0,ncol(cormat)),
cormat),N,dimnames=list(1:N,colnames(cormat))))

### generating Y
data.Y <- simResponseReg(Exp=data.X, Nexp=NPred, beta=betaMean, R2=R2*NPred,
CorrLim=corr)
beta <- cbind(var=gsub("[:]","*",names(data.Y$beta)),val= data.Y $beta)
rownames(beta) <- gsub("á","a",rownames(beta))

################################################################################
##                                   APPLYING THE REGRESSION-BASED METHODS
################################################################################

####################### EWAS and EWAS-MLR results
library(Matrix)
library(MASS)
library(parallel)
# parameters for the EWAS
mlcorr <- "BY" # the method to correct for multiple testing (amongst Bon=Bonferroni,
BH=Benjamini-Hochberg, BY=Benjamini-Yakutieli)
res <- EWAS(resp=data.Y$resp, Exp=data.X, beta=beta, interaction=1)
resEWAS  <- testReg(Exp=data.X, outc=data.Y, p.values= res[,c("var","pVal")], intTest=1,
corr=mlcorr, par=1, Nperm=NA, lambda=NA, interaction=1,EWASonly=FALSE)
RES <- merge(res[,c("var","val","Est")],data.frame(cbind(var=colnames(resEWAS)[-
(1:2)],t(resEWAS[c(1,2,3),-(1:2)]))),by="var",all.y=TRUE)

####################### DSA
library(DSA)
# this only exists for R version up to 2.15, and the package can be downloaded from
# http://www.stat.berkeley.edu/~laan/Software/
resDSA <- DSAreg(Exp= data.X, resp= data.Y$resp, maxsize = 4, maxsumofpow =1,
maxorderint = 1)
RES <- merge(RES,resDSA$coef,by='var',all.x=TRUE)
RES[,ncol(RES)] <- as.numeric(as.character(RES[,ncol(RES)]))
RES[which(is.na(RES[,ncol(RES)])), ncol(RES)] <- 0
colnames(RES)[ncol(RES)] <- "DSA"

####################### ENET
library(glmnet)
resENET <- EvaluateModel.cv.glmnet(x=as.matrix(data.X),y=data.Y$resp[,1])
RES <- merge(RES,cbind(var=colnames(data.X),t(resENET[,-(1:2)])),by='var',all.x=TRUE)
```

```
colnames(RES)[ncol(RES)+1-nrow(resENET):1] <-
paste(resENET[,1],"_",resENET[,2],sep="")

###################### R2GUESS
library(R2GUESS)
# parameter for the R2GUESS function
cutoffModels <- 0.01
if(NPred>=5){  Egam <- NPred+2; Sgam <- 5
}else{ Egam <- 3;  Sgam <- 3  }

ResMPPI <- BestModels <- NULL
Res <- NULL
CurrentSeed <- 100000+NPred*1000
#Params to enter R2GUESS
path.par <- paste(system.file("extdata", package="R2GUESS"),"/",sep="")
file.par <- "Par_file_example_Hopx.xml"
root.file.output <- paste("R2GUESS_",NPred,"_pred_Scen_Simul_" ,sep="")
model <- R2GUESS(dataY= as.data.frame(data.Y$resp),dataX= data.X, getwd(),
getwd(),path.par,
                        file.par=file.par,nsweep=5000,burn.in=1000,label.X=colnames(data.X),
                        root.file.output=root.file.output, time=TRUE, top=100,history=TRUE,
Egam=Egam,
                   Sgam=Sgam,
nb.chain=3,conf=NULL,cuda=FALSE,MAP.file=NULL,seed=CurrentSeed)

MPPI <-
read.table(paste(getwd(),"/",root.file.output,'_output_marg_prob_incl.txt',sep=''),header=T)
ResMPPI <- rbind(ResMPPI,MPPI[,2])
colnames(ResMPPI) <- colnames(data.X)
idx <- which(model$BestModels$postProb>cutoffModels)
bm <- do.call(cbind, model$BestModels)
bm <- bm[idx,c("Rank", "ModeSize", "postProb", "modelName")]

resGUESS <- EvaluateModel.R2GUESS(x=as.matrix(data.X),y=data.Y$resp[,1],
MPPI=ResMPPI)
colnames(resGUESS)[-(1:2)] <- colnames(data.X)
RES <- merge(RES,cbind(var=colnames(resGUESS),t(resGUESS)),by='var',all.x=TRUE)
colnames(RES)[ncol(RES)+1-nrow(resGUESS):1] <- paste(resGUESS [,1],"_",resGUESS
[,2],sep="")

###################### sPLS
library(spls)
resSPLS <- EvaluateModel.cv.spls(x=as.matrix(data.X),y=data.Y$resp[,1])
colnames(resSPLS)[-(1:2)] <- colnames(data.X)
RES <- merge(RES,cbind(var=colnames(resSPLS),t(resSPLS)),by='var',all.x=TRUE)
colnames(RES)[ncol(RES)+1-nrow(resSPLS):1] <- paste(resSPLS[,1],"_",
resSPLS[,2],sep="")
```

Excel File Table S1. Matrix of all Pearson pairwise correlation coefficients in Σ, the semi-definite matrix the closest to the correlation matrix derived from the INMA cohort (see Supplemental Code and Data Zip File for this table).

DOI: 10.1289/EHP172

Table S2. Sensitivity and FDP obtained when augmenting the list of variables selected by a method with variables that are correlated (in absolute value) to those variables above some threshold α (with N an average number of variables in total), with α values varying in the range 0.6,0.7,0.8,0.9,1 (1 standing for the scenarios set 1). The table also includes results for the hypothetical situation in which we had a perfect model (oracle) to illustrate the limitations of having highly correlated data. Results are given for scenarios with k=0,1,2,3,5,10,25 true predictors.

		k=1			k=2			k=3			k=5			k=10			k=25		
Method	α	N	sens	FDP	N	sens	FDP	N	sens	FDP	N	sens	FDP	N	sens	FDP	N	sens	FDP
ORACLE	1	1,0	100%	0%	2,0	100%	0%	3,0	100%	0%	5,0	100%	0%	10,0	100%	0%	25,0	100%	0%
	0,9	1,8	100%	22%	3,3	100%	26%	5,0	100%	30%	8,5	100%	33%	16,4	100%	36%	38,7	100%	35%
	0,8	2,7	100%	43%	5,8	100%	55%	7,7	100%	54%	13,3	100%	58%	26,7	100%	61%	58,3	100%	57%
	0,7	4,7	100%	56%	10,1	100%	69%	12,6	100%	68%	21,5	100%	73%	43,0	100%	75%	84,3	100%	70%
	0,6	6,9	100%	65%	15,3	100%	79%	19,5	100%	79%	31,1	100%	82%	60,5	100%	83%	108,7	100%	77%
EWAS	1	11,0	97%	67%	33,3	98%	87%	44,7	97%	89%	81,0	97%	93%	131,3	94%	93%	175,4	90%	87%
	0,9	11,5	97%	68%	34,5	98%	87%	46,5	97%	90%	83,6	98%	93%	133,9	94%	93%	177,7	91%	87%
	0,8	14,5	98%	69%	40,3	98%	89%	54,0	97%	91%	92,4	98%	94%	141,0	94%	93%	181,7	91%	87%
	0,7	18,5	98%	71%	48,5	98%	90%	63,8	97%	92%	102,9	98%	95%	148,0	94%	94%	184,7	91%	88%
	0,6	23,6	98%	74%	56,7	98%	92%	72,8	97%	94%	112,9	98%	95%	156,8	95%	94%	190,7	92%	88%
EWAS-MLR	1	1,3	55%	30%	2,6	42%	52%	3,4	44%	52%	4,9	32%	59%	8,6	18%	73%	10,4	7%	80%
	0,9	1,8	57%	35%	3,2	42%	54%	4,3	44%	56%	6,3	34%	61%	11,5	19%	77%	14,6	9%	82%
	0,8	2,9	61%	40%	5,8	48%	60%	7,1	48%	65%	10,3	38%	69%	19,1	24%	81%	23,3	12%	84%
	0,7	4,3	63%	43%	10,5	55%	65%	12,5	53%	71%	17,8	42%	76%	30,3	29%	85%	36,0	17%	85%
	0,6	6,7	65%	50%	15,1	58%	71%	18,5	56%	77%	25,8	45%	81%	42,1	35%	87%	48,1	23%	85%
ENET	1	0,4	29%	11%	1,9	60%	25%	3,5	79%	26%	7,8	74%	49%	18,7	77%	57%	45,3	80%	56%
	0,9	0,7	36%	12%	2,8	66%	32%	5,4	86%	39%	11,5	84%	59%	25,9	86%	65%	60,4	89%	63%
	0,8	1,2	37%	21%	5,2	68%	49%	8,3	88%	57%	18,7	87%	73%	41,0	91%	76%	85,8	94%	72%
	0,7	1,8	38%	24%	8,8	71%	58%	13,9	89%	69%	30,4	91%	82%	62,7	93%	84%	113,7	96%	79%
	0,6	2,5	38%	27%	12,7	73%	65%	20,6	90%	78%	41,7	91%	87%	82,3	94%	88%	136,4	96%	82%
sPLS	1	4,3	90%	40%	4,8	87%	38%	9,6	91%	37%	16,7	80%	53%	31,9	79%	63%	119,6	90%	79%
	0,9	4,9	97%	43%	5,9	92%	46%	10,9	94%	48%	19,0	84%	62%	35,8	82%	67%	125,4	92%	80%
	0,8	6,5	98%	56%	9,1	94%	63%	14,2	95%	63%	25,6	88%	74%	48,3	87%	76%	138,8	94%	82%
	0,7	8,9	98%	63%	14,3	96%	74%	19,7	96%	73%	36,3	93%	81%	66,8	91%	83%	153,3	95%	84%
	0,6	12,1	98%	71%	20,4	97%	82%	27,4	96%	82%	48,1	94%	87%	85,5	94%	87%	168,0	97%	85%
GUESS	1	1,7	91%	30%	3,3	92%	34%	4,8	92%	33%	9,0	89%	45%	17,3	85%	48%	39,3	82%	46%
	0,9	2,2	95%	34%	4,2	93%	43%	5,8	93%	42%	11,1	91%	53%	20,8	87%	55%	49,5	87%	55%
	0,8	3,3	98%	48%	7,2	96%	62%	8,8	95%	60%	17,6	94%	69%	32,5	91%	70%	71,8	93%	67%
	0,7	5,2	99%	59%	11,8	97%	73%	14,2	96%	71%	27,6	95%	79%	49,8	94%	79%	98,3	96%	75%
	0,6	7,8	99%	68%	17,2	98%	81%	21,2	96%	80%	39,1	96%	85%	67,8	96%	85%	122,5	97%	80%
DSA	1	1,4	79%	29%	2,1	80%	21%	3,1	77%	24%	5,0	71%	27%	9,7	66%	31%	24,6	65%	33%
	0,9	2,3	90%	35%	3,3	87%	35%	5,1	86%	40%	8,5	80%	47%	15,7	76%	48%	37,8	76%	49%
	0,8	3,5	95%	49%	5,9	90%	57%	7,9	91%	57%	13,5	86%	65%	25,0	84%	64%	58,2	86%	62%
	0,7	5,5	97%	59%	9,9	93%	69%	12,9	93%	70%	22,5	90%	77%	40,6	88%	77%	85,6	92%	73%
	0,6	8,1	97%	68%	14,9	94%	78%	19,6	94%	80%	32,7	92%	84%	57,6	92%	83%	111,0	95%	78%

DSA: Deletion/substitution/addition; ENET: Elastic net; EWAS: Environment-wide association study; EWAS-MLR: EWAS-Multiple Linear Regression; GUESS: Graphical Unit Evolutionary Stochastic Search; sPLS: Sparse partial least-squares.

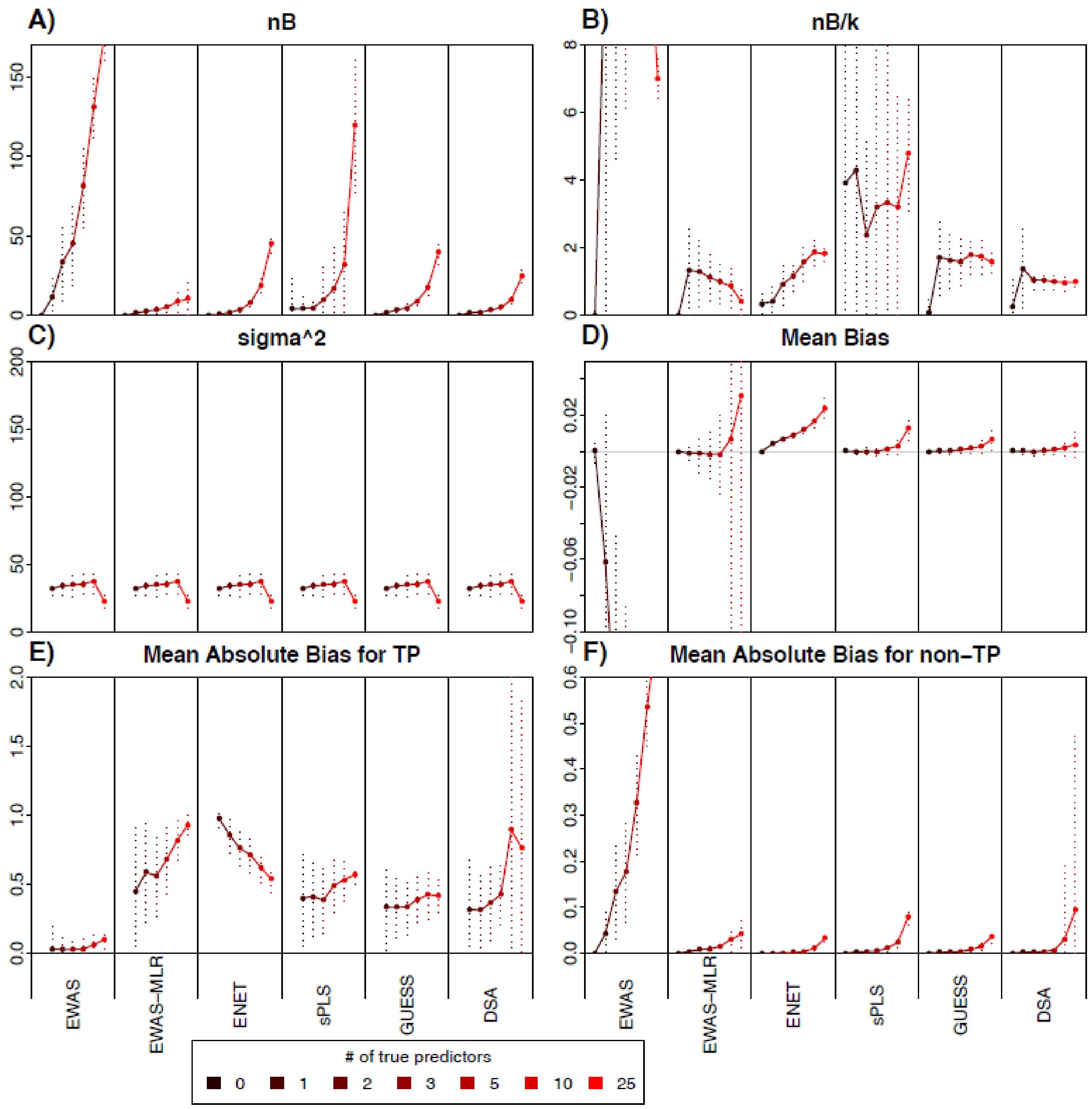

Figure S2. Additional parameters characterizing the performances of the statistical methods for scenarios set 1. Model performances are summarized by n_B the number of variables selected by the method (A), the ratio n_B/k with k the number of true predictors (=n_B when $k = 0$) (B), the error variance σ^2 (C), the mean bias (all coefficient values being equal to 1) (D), the mean absolute bias computed over the true predictors (E) and over the other variables that are not true predictors (F). For each scenario defined by a number of true predictors varying from 0 to 25, statistics over the 100 runs are summarized by their mean (dot) and the variability of each statistics is summarized using the one standard error both ways from the average value (vertical dotted line). DSA: Deletion/substitution/addition; ENET: Elastic net; EWAS: Environment-wide association study; EWAS-MLR: EWAS-Multiple Linear Regression; GUESS: Graphical Unit Evolutionary Stochastic Search; sPLS: Sparse partial least-squares; TP: True Predictors.

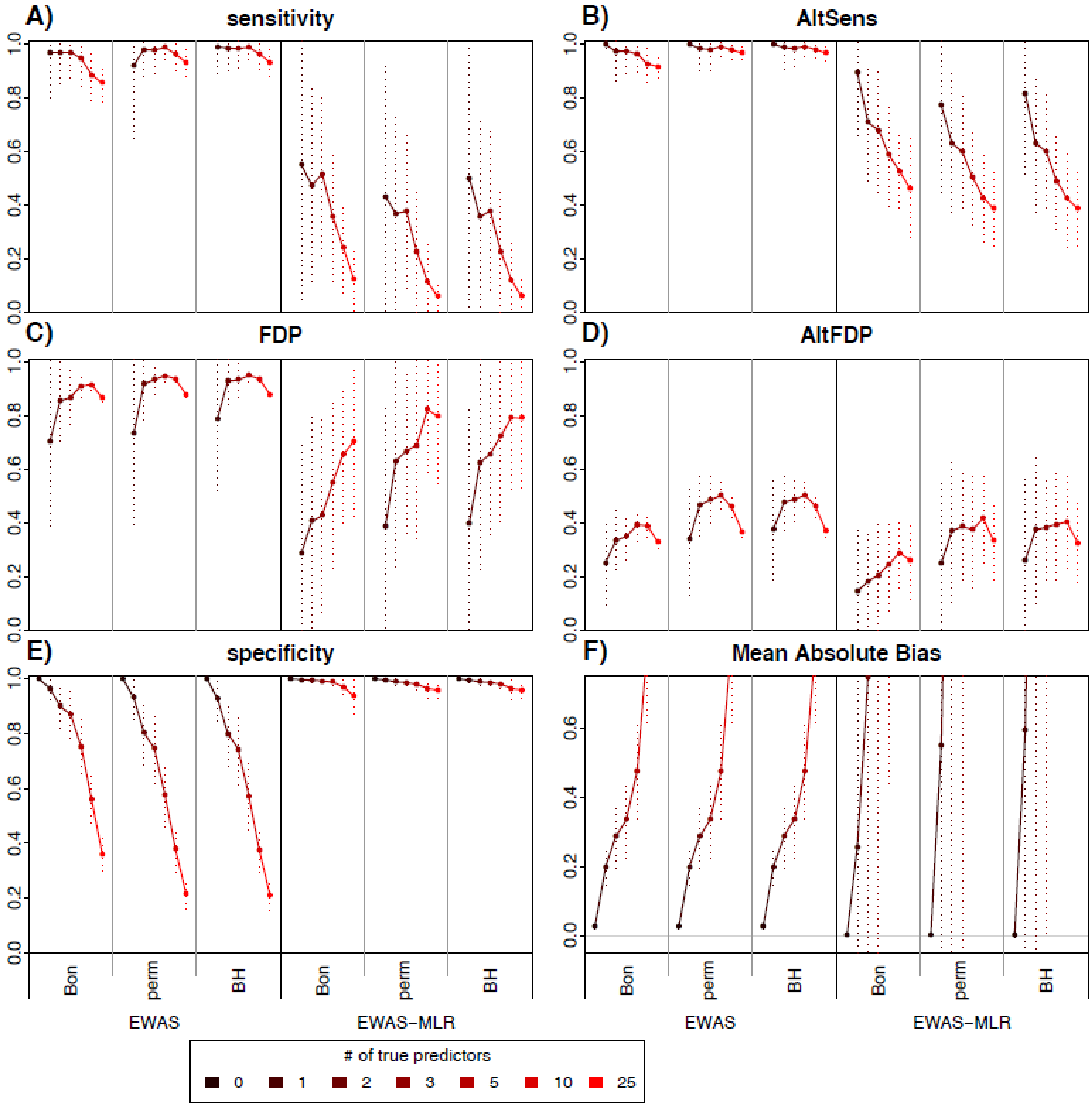

Figure S3. Performances of the statistical methods for scenarios set 1 using alternative multiple hypothesis testing corrections for the EWAS and EWAS-MLR methods: the Bonferroni (Bon), permutation (perm) and Benjamini and Hochberg (BH) corrections. For each scenario defined by a number of true predictors varying from 0 to 25, statistics over the 100 runs are summarized by their mean (dot) and the variability of each statistics is summarized using the one standard error both ways from the average value (vertical dotted line). EWAS: Environment-wide association study; EWAS-MLR: EWAS-Multiple Linear Regression.

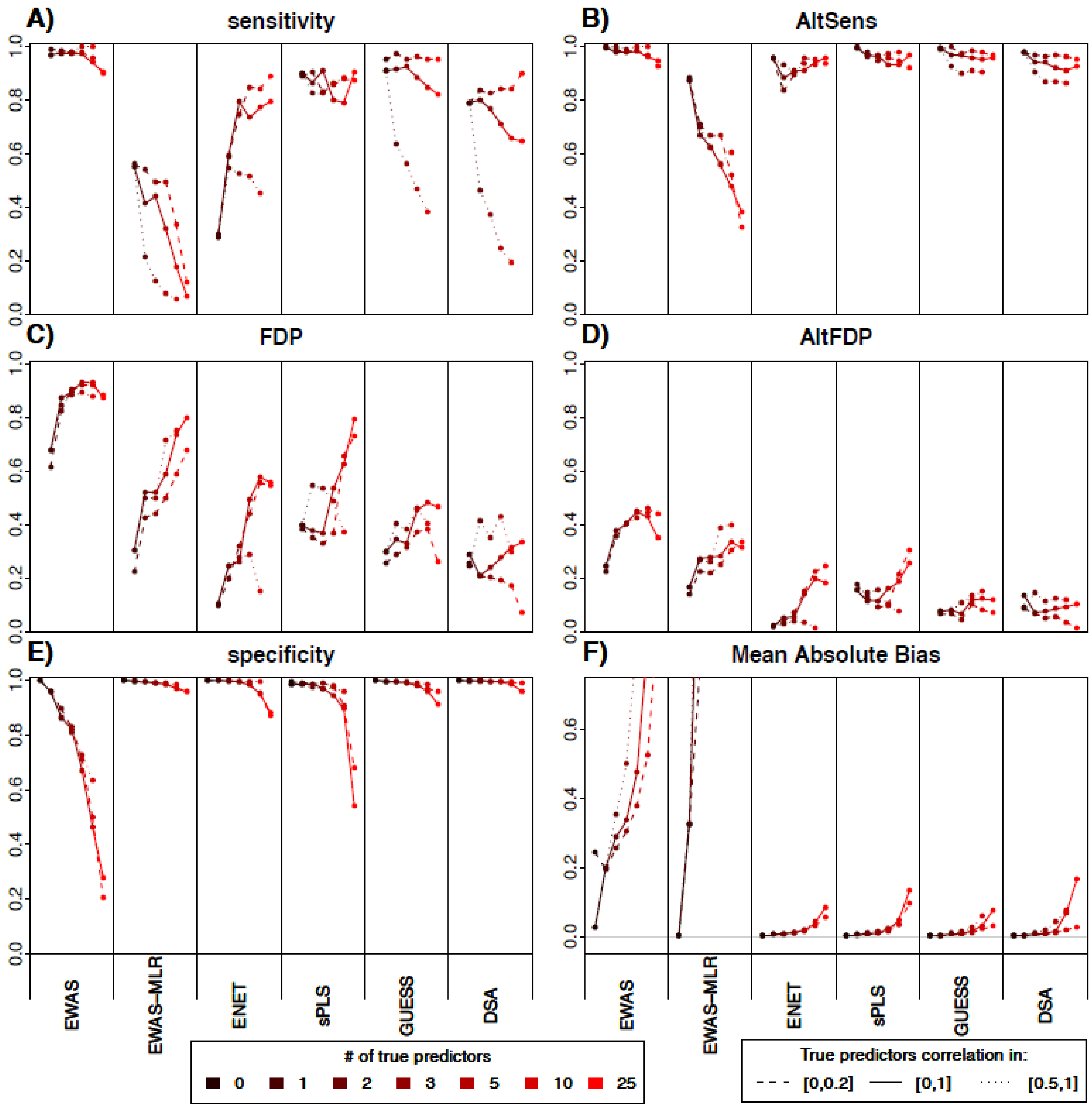

Figure S4. Performances of the statistical methods according to the amount of correlation between the true predictors. The full line connects the results for scenarios with correlations between the true predictors (in absolute values) in the [0,1] range (scenarios set 1); the dashed line in the [0,0.2] range (scenarios set 2) and the dotted line in the [0.5,1] range (scenarios set 3). For each scenario defined by a number of true predictors varying from 0 to 25 (to 10 for set 3, Σ not containing 25 exposures that are correlated at a level >0.5), statistics over the 100 runs are summarized by their mean (dot). DSA: Deletion/substitution/addition; ENET: Elastic net; EWAS: Environment-wide association study; EWAS-MLR: EWAS-Multiple Linear Regression; GUESS: Graphical Unit Evolutionary Stochastic Search; sPLS: Sparse partial least-squares.

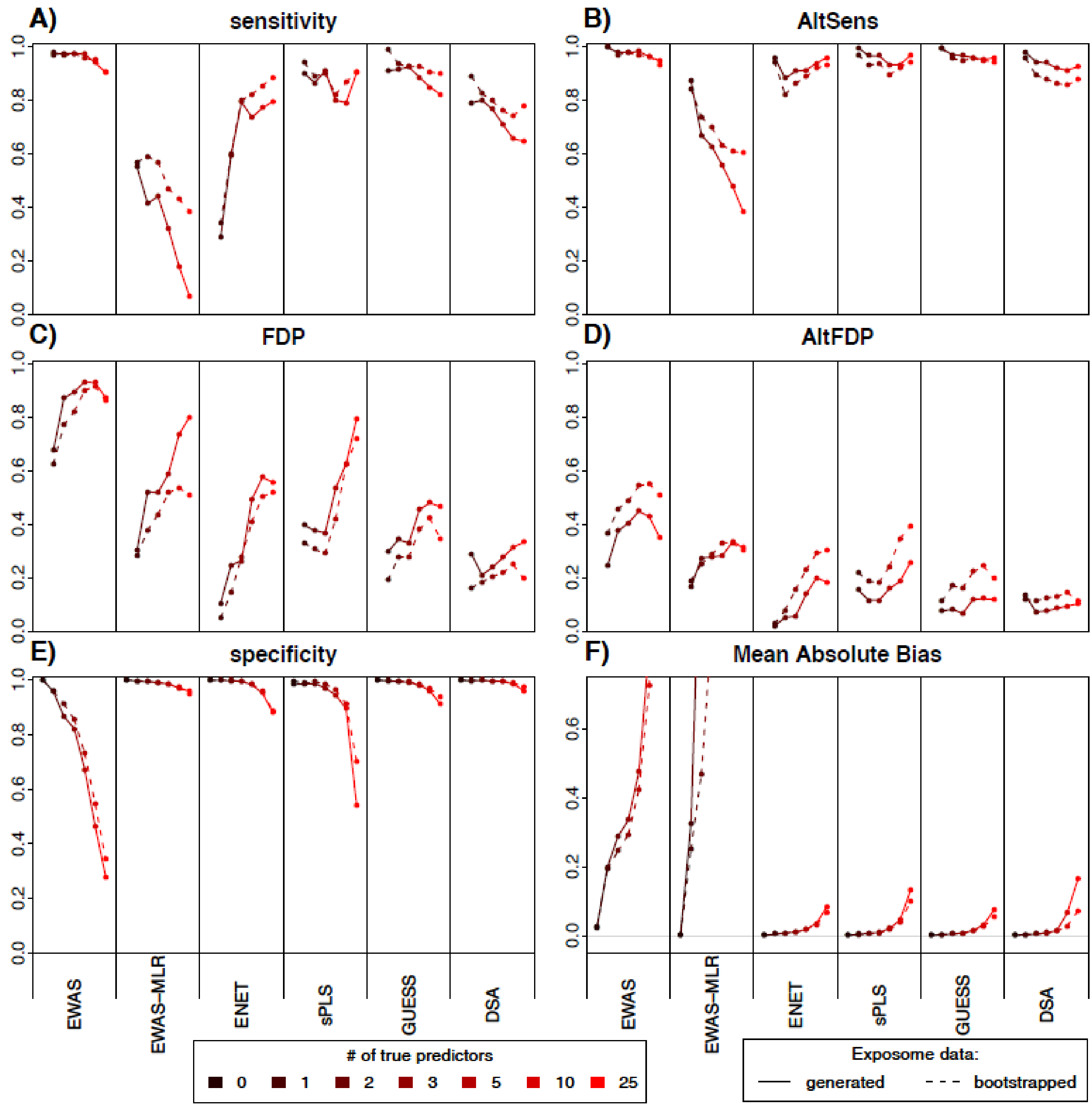

Figure S5. Performances of the statistical methods when deviating from the assumption of normally distributed exposures. The full line connects the results for scenarios set 1 (exposures generated from a normal distribution); the dotted line corresponds to the same scenarios being tested on exposure data bootstrapped from the INMA environmental data (scenarios set 6). For each scenario defined by a number of true predictors varying from 0 to 25, statistics over the 100 runs are summarized by their mean (dot). DSA: Deletion/substitution/addition; ENET: Elastic net; EWAS: Environment-wide association study; EWAS-MLR: EWAS-Multiple Linear Regression; GUESS: Graphical Unit Evolutionary Stochastic Search; sPLS: Sparse partial least-squares.

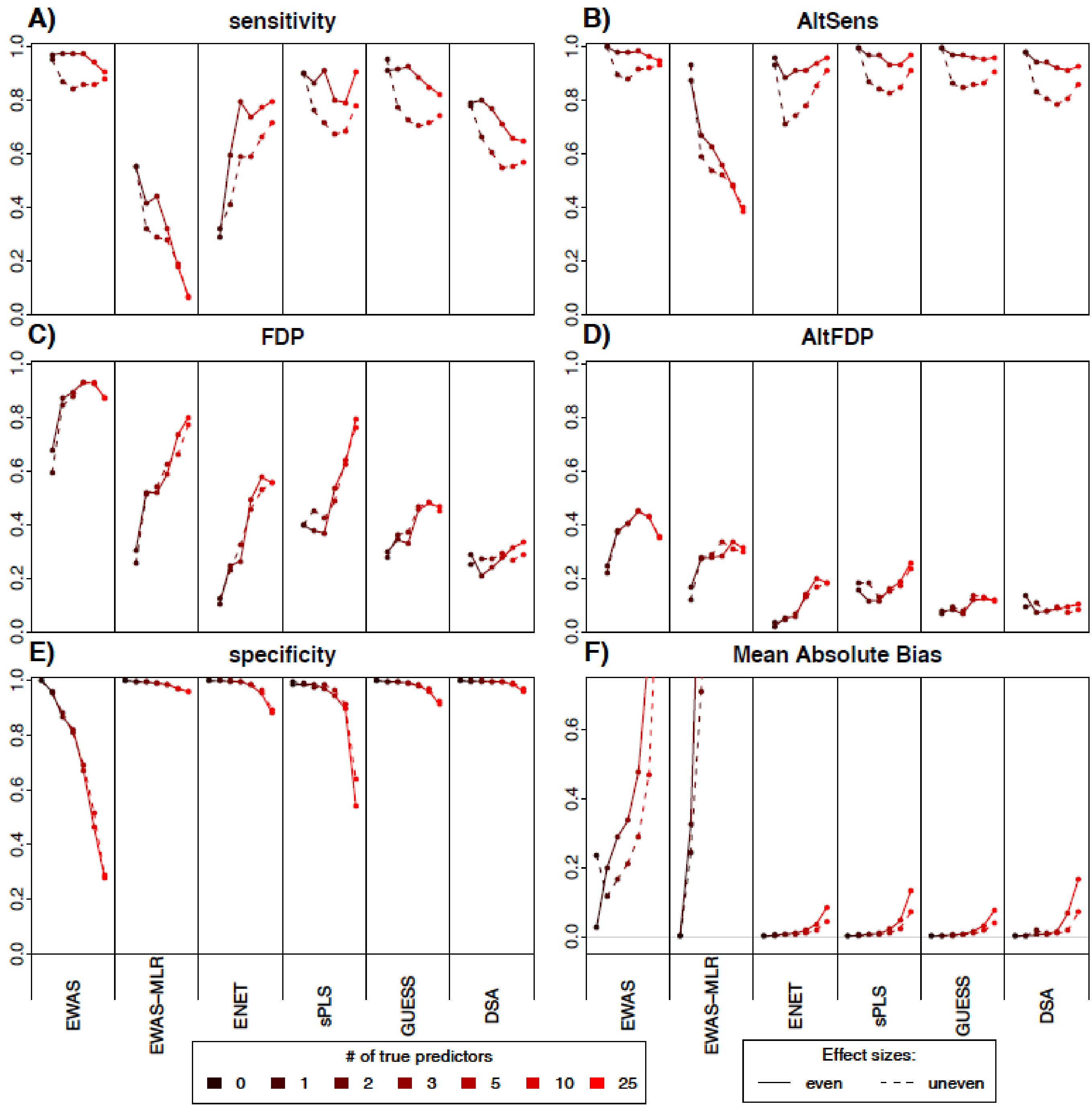

Figure S6. Performances of the statistical methods for varying effect sizes among true predictors. The full line connects the results for scenarios set 1 (all effect sizes equal to 1); the dotted line corresponds to the same scenarios except with effect sizes generated from a uniform distribution in [0.5,1.5] (scenarios set 7). For each scenario defined by a number of true predictors varying from 0 to 25, statistics over the 100 runs are summarized by their mean (dot). DSA: Deletion/substitution/addition; ENET: Elastic net; EWAS: Environment-wide association study; EWAS-MLR: EWAS-Multiple Linear Regression; GUESS: Graphical Unit Evolutionary Stochastic Search; sPLS: Sparse partial least-squares.
